# Identification of the earliest collagen- and plant-based coatings from Neolithic artefacts (Nahal Hemar cave, Israel)

**DOI:** 10.1038/srep31053

**Published:** 2016-08-09

**Authors:** Caroline Solazzo, Blandine Courel, Jacques Connan, Bart E. van Dongen, Holly Barden, Kirsty Penkman, Sheila Taylor, Beatrice Demarchi, Pierre Adam, Philippe Schaeffer, Arie Nissenbaum, Ofer Bar-Yosef, Michael Buckley

**Affiliations:** 1 Museum Conservation Institute, Smithsonian Institution, Suitland, MD 20746, USA; 2 Laboratoire de Biogéochimie Moléculaire, Institut de Chimie de Strasbourg, UMR 7177, Université de Strasbourg, 67200 Strasbourg, France; 3 23 rue Saint-Exupéry, 64000 Pau, France; 4 School of Earth, Atmospheric and Environmental Sciences, Williamson Research Centre for Molecular Environmental Science, University of Manchester, Manchester, M13 9PL, United Kingdom; 5 BioArCh, Department of Chemistry, University of York, York, YO10 5DD, United Kingdom; 6 Department of Earth and Planetary Sciences, Weizmann Institute of Science, Rehovot 76100, Israel; 7 Department of Anthropology, Harvard University, Cambridge, MA 02138, USA; 8 Faculty of Life Sciences, Manchester Institute of Biotechnology, University of Manchester, Manchester, M1 7DN, United Kingdom

## Abstract

Mortuary practices in human evolution record cognitive, social changes and technological innovations. The Neolithic Revolution in the Levant was a watershed in this domain that has long fascinated the archaeological community. Plaster modelled skulls are well known at Jericho and several other Neolithic sites, and in Nahal Hemar cave (Israel, ca. 8200 −7300 cal. BC) excavations yielded six unique human skulls covered with a black organic coating applied in a net pattern evoking a headdress. This small cave was used as storage for paraphernalia in the semi-arid area of the Judean desert and the dry conditions preserved other artefacts such as baskets coated with a similar dark substance. While previous analysis had revealed the presence of amino acids consistent with a collagen signature, in the present report, specific biomarkers were characterised using combined proteomic and lipid approaches. Basket samples yielded collagen and blood proteins of bovine origin (*Bos* genus) and a large sequence coverage of a plant protein charybdin (*Charybdis* genus). The skull residue samples were dominated by benzoate and cinnamate derivatives and triterpenes consistent with a styrax-type resin (*Styrax officinalis*), thus providing the earliest known evidence of an odoriferous plant resin used in combination with an animal product.

Mortuary practices and substances employed in rituals are considered as recording the diversity of social and technological evolution of past societies. Rich archaeological evidence from the Late Pleistocene and most of the Holocene lack the written documents reporting the underlying symbolism and its place in the ideology of the users of these techniques. Historical cases include artefacts such as vases containing myrrh^1^, ceramics with birch bark tar^2^, incense burners with frankincense^3^, and mummies with remains of embalming organic materials like coniferous or *Pistacia* resins^4^,^5^. Although the treatment of the dead began during the Paleolithic period, the transition from foraging to farming, generally known as the Neolithic Revolution in the Levant, resulted in new social arrangements, a suite of technological inventions, and an unexpected diversity in mortuary practices and creations of human figures. The best known are the plaster modelled skulls and human statues^6^,^7^,^8^,^9^,^10^, cultural features of the Pre-Pottery Neolithic B (PPNB) period in the Near East (ca. 8600 to 6200 BC). The skulls were found in Jericho, Ain Ghazal, Kefar HaHoresh, Yiftahel, Beisamoun and Tel Ramad and considered to have been involved in ritual practices and ancestors’ cult. The plaster statues found only in Jericho and Ain Ghazal, that possibly stood in the village’s shrine, were recovered from special dumping pits after they went out of use^9^,^10^.

Here we report, in the context of examining the new Neolithic treatments of the dead, a unique set of six decorated skulls found in Nahal Hemar cave in the Judean Desert (Fig. 1a)^11^,^12^,^13^. The cave is a small, dark chamber probably used for storage of paraphernalia for planned ceremonies. The skulls were found in the lower layer (layer 4), where pieces of twined linen including a head gear and a napkin, baskets, painted wooden beads, a sickle, bone tools and a special type of flint blades, were found in 1982–3 and radiocarbon dated to 8210–7300 cal. BC (Table 1)^11^ placing them in the Middle Pre-Pottery Neolithic B period (see SI). The skulls coating was directly dated to 8920–8430 cal. BC (Table 2). Contrary to the skulls modelled with lime plaster^8^, those discovered in Nahal Hemar cave were treated differently. The cranial vault was covered with a thin layer of a brown-black substance and subsequently adorned with a similar material applied in a net pattern (Fig. 1b). In addition to these skulls, other well-preserved organic finds include fragments of baskets that were also coated with a black substance, as well as loose lumps of coating-like residue.

The origin of the people responsible for the Nahal Hemar artefacts is still unknown. Most of the finds including a stone mask, and especially the plant remains, were brought from the hilly region characterised by the Mediterranean vegetation belt. Similar stone masks were found in the general area south of Jerusalem^14^. Evidence from the carnelian bead indicates that the lapidary technique employed in piercing the rock was common in the northern Levant^15^. The proposal that the finds may have been made by a local desert tribe who favoured the cave for burial of cultic objects was suggested when the black substance lining of the skulls and baskets was first believed to correspond to bitumen, a locally accessible material^16^ well-known for its water-resistant properties^17^. Examples of bitumen use have been found in the earlier PPNA age Gilgal I site in the Jordan Valley, dated to ca. 9,200 BC^18^,^19^. However, pyrolysis and amino acid analysis (by Mary Ann Gawinowicz, Columbia University, 1994) on a basket sample (#448, Table 2) and a flake (#672) of the loose coating-like material revealed a protein origin characterised by a collagen signature^18^,^20^, which led to the suggestion of an early use of collagen as binding material (referred in the early studies as “glue”) albeit with a tenuous link between the artefact types. The preparation and use of collagen-based material so early in prehistory was remarkable given that, in the area, the processing of collagen into glue only appears much later during the 3^rd^ millennium BC in Egypt where it was used on furniture items and as a painting binder^21^. Without further analysis or other Neolithic examples of glue production, the nature of the collagen was speculative. Modern analytical techniques have advanced greatly over the past two decades, particularly with respect to their applications to archaeological residue analysis. The exceptional nature of the Nahal Hemar archaeological site warranted a re-examination of these organic coating substances (Table 2) using some of these modern techniques, particularly protein analysis using proteomic methodologies and lipid analysis by gas chromatography mass spectrometry (GC/MS) and pyrolysis GC/MS (py-GC/MS) to identify the source of the collagen and detect potential additional components.

## Results

### Revisiting the collagen signatures

**Figure 2:**
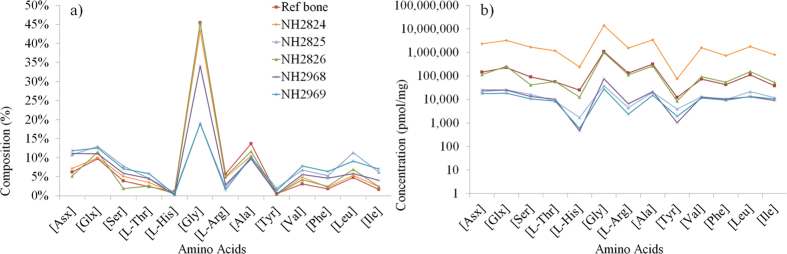
(**a**) Amino acid composition and (**b**) concentrations of the amino acids in the Nahal Hemar samples and in a reference bone sample (protein composition dominated by collagen). Note: the presence of amino acids in NH2969, not directly in contact with the skull, rules out the possibility of a contamination from skin and bones from the skull.

The RP-HPLC distribution of the amino acids obtained after hydrolysis of the basket and skull coating residues (Fig. 2, Table S1) was characterised by a high relative proportion of glycine (Fig. 2a) typically observed in collagen-dominated tissues such as bone^22^. Proline and hydroxyproline were not detected using this RP-HPLC method (as they are secondary amino acids and therefore not derivatised), but they were identified through GC/MS analyses (partial gas chromatograms shown in Figs S1–3). Amino acid concentrations in the basket subsample NH2825 and the skull coating subsamples were low. The basket subsample NH2826 had a concentration in amino acids typical of bone collagen (see bone reference Fig. 2b), but was still 15 times lower than NH2824, taken from a fragment with resin-like aspect found in a superior layer, previously identified as collagen in Connan *et al.*^18^. This sample served as a reference to assess the potential of collagen preservation in the cave and likely comes from gelatinised bone or skin (see Discussion). In addition, the Py-GC/MS data for this reference sample, shown in SI (Table S2 and Fig. S4A), revealed products of proline- and hydroxyproline-containing structural moieties from collagen proteins comprising pyrroline, pyrrole, diketopyrrole, and the 2,5-diketopiperazine derivatives (cyclic compounds of two amino acids) of Pro-Ala, Pro-Gly, Pro-Lys, and Pro-Pro. The predominance of these compounds supports a mostly pure collagen origin, and presence of peptides.

Of the four residue samples analysed these compounds were found only in basket sample NH2826 (Fig. S4C), but in lower relative quantities if compared to NH2824. A decrease in the relative quantity of these dimerization products has been associated with a disruption of the polymer chains or collagen degradation^23^. In the other basket sample NH2825, multiple pyridine and pyrrole derivatives (Table S2, Fig. S4B) were identified (in collagen these mainly correspond to pyrolysis products of Pro, Hyp and Ala)^23^ but not the diketopyrrole and diketopiperazine derivatives.

### Taxonomic sources of animal proteins

**Figure 3:**
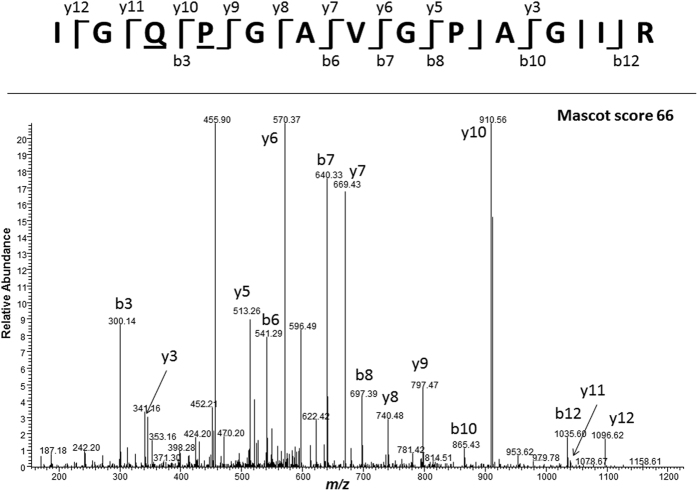
Tandem MS spectrum of IGQPGAVGPAGIR, M = 1208.651, with deamidation in Q_3_ and hydroxylation in P_4_ to Hyp from collagen alpha-2(I) chain CO1A2_BOVIN, identified by Mascot, from the basket sample NH2826.

The more informative proteomic analyses identified the expected collagen type I (alpha-1 and -2) protein in both NH2826 and the reference flake NH2824 (Table 2) with respectively 25% and 61% percentage coverage for collagen α1(I) and 33% and 48% for collagen α2(I) (Tables S3 and S4) in agreement with the less well-preserved collagen observed in this basket sample compared to the flake. Both samples could be confidently identified as bovine (i.e., *Bos* given the geographical context), due to the presence of the species biomarker IGQPGAVGPAGIR from collagen α2(I)^24^. In NH2824, the peptide was identified at M = 1191.672 (M = 1207.667 with the proline residue P_4_ modified to hydroxyproline) and at M = 1192.656 (Fig. S5) and 1208.651 with additional deamidation of the glutamine Q_3_. In NH2826, the peptide was identified in its deamidated form only (Fig. 3).

In NH2824, the presence of blood was affirmed by the identification of bovine haemoglobins alpha and beta (44% and 47% percentage coverage respectively) and other major plasma proteins such as fibrinogen proteins (Table 3).

In addition to collagen, several other extracellular matrix (ECM) proteins, all belonging to the family of small leucine-rich repeat proteoglycans (SLRPs) and playing a role in the growth and organisation of collagen fibrils, were found. However the different types of SLRP proteins identified in NH2824 and NH2826 might be indicative of a different origin for the collagen, with the distribution in NH2826 more consistent with bone SLRPs. In NH2826, the SLRPs were characterised by fibromodulin (10% coverage) and biglycan (20% coverage) associated with collagen fibrillogenesis and by the aggrecan core protein (4% coverage) found in cartilage, while in NH2824 decorin, lumican and mimecan were found (Table 3, S3): decorin (26%) has a role in matrix assembly through interaction with type 1 collagen^25^, lumican (21%) is another collagen-binding protein participating in collagen fibril assembly^26^ mainly expressed in cornea, dermis and tendon^27^, and mimecan (17%) is known to influence collagen fibril diameter and interfibrillar spacing^28^.

### Taxonomic sources of plant biomolecules

In the other basket (NH2825) and skull (NH2968 and NH2969) samples where the amino acid concentration was low, no collagen peptides were identified (Tables S4 and S5). In the basket sample NH2825 plant triterpenoids and sterols as well as plant pyrolysis products (furanones, guaiacol, cresol) were identified instead (Table S2, Fig. S4B) and the presence of a plant protein was confirmed with the identification of charybdin (percentage coverage of 55%, Table S4), a 29-kDa type 1 ribosome-inactivating protein (RIP) that was characterised and named in Touloupakis *et al.*^29^ in the bulb of the white variety of *Charybdis maritima*. Two peptides were found to be unique to charybdin using Blast against all entries (http://blast.ncbi.nlm.nih.gov/): LTGQTYTDFIK (Fig. S6) and SLIVVSQMFCEATR (Fig. S7). Charybdis (or Drimia), known by its common name as sea squill (or sea onion), is native of the Mediterranean coastal regions and is represented by several species^30^ (see SI for the taxonomy of Charybdis/Drimia), of which charybdin has to date only been sequenced from *C. maritima*. It is therefore possible that the charybdin protein identified in the basket originates from a *Charybdis* species not yet sequenced.

**Figure 4:**
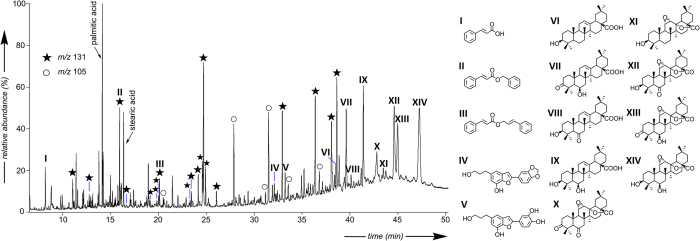
Gas chromatogram of the total lipid extract from the Neolithic skull (NH2968) found in the Nahal Hemar cave. Cinnamic (*m/z* 131) and benzoic (*m*/*z* 105) ester derivatives are identified respectively by filled stars and open circles. Alcohols are detected as acetates and acids as methyl esters.

**Figure 5:**
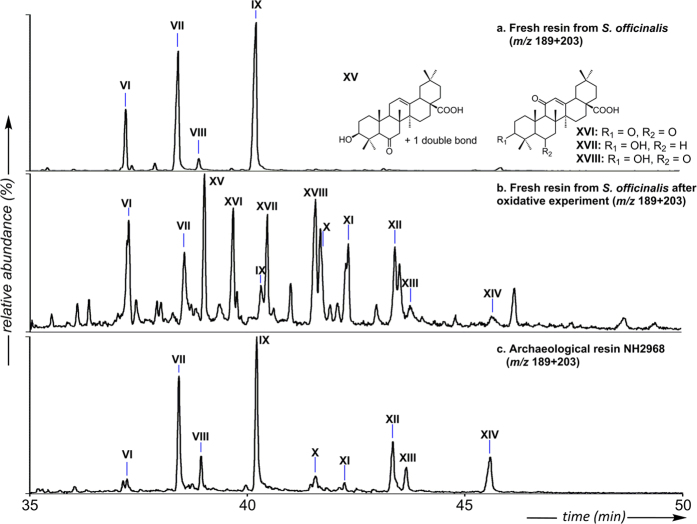
Partial GC/MS chromatogram showing the distribution of triterpenoids presenting fragment(s) at *m*/*z* 189 + 203 in (**a**) the fresh resin of *S. officinalis* collected at Yodfat (Israel), (**b**) the fresh resin after 13 hours of oxidative treatment, (**c**) the archaeological skull sample NH2968. Alcohols are detected as acetates and acids as methyl esters. Compound numbers **VI–XIV** refer to the structures reported on Fig. 4.

In contrast to the lipid extract from the collagenous sample NH2824, which is extremely low (0.5%, Table S6), the lipid extracts of the skull coating samples (NH2968 and NH2969) represent a significant part of the investigated materials (>67%, Table S6), suggesting that the latter comprise additional predominant solvent-soluble constituent(s). GC/MS analyses of these total lipid extracts (Fig. 4 and S8) revealed a botanical origin of this component, which is in agreement with py-GC/MS results (Fig. S4D,E). Indeed, besides the presence of phenolic compounds (phenols, guaiacols and syringols), which are typical of lignin^31^, both samples contained a number of aromatic ester derivatives of cinnamic and benzoic acids, suggesting the contribution of a balsam, and more precisely, of a styrax-type resin. In the Mediterranean region this resin is exclusively produced in response to an incision of two types of trees: *Styrax officinalis* (*Styracaceae* family) and *L. orientalis* (*Hamamelidaceae* family). Both were known and utilised in antiquity, for example as an incense, medicinal or cosmetic product^32^. While their modern distribution (South of Europe and the Middle East^33^ for the former, and the South of Turkey, Cyprus and Rhodes^34^ for the latter) would favour *S. officinalis* as the source of the archaeological resin, both were considered as a potential match. GC/MS investigation of fresh *S. officinalis* (Fig. S9) and *L. orientalis* resins revealed the predominance of cinnamyl cinnamate (III, Fig. 4) in the former and cinnamyl cinnamate associated to 3-phenylpropanyl cinnamate in the latter. However, discrimination between these two botanical sources in the archaeological coating was not possible based on the distributions of cinnamate and benzoate derivatives, especially since the distribution of these derivatives may have been altered upon ageing. This would be in agreement with the detection of numerous cinnamate and benzoate derivatives (presence of characteristic fragments at *m*/*z* 105 or 131) specifically occurring among the lipids from the archaeological samples. However, these compounds could not be firmly identified based on their mass spectra and it is proposed that they correspond to alteration or condensation products of cinnamates and benzoates. Subsequently the chemotaxonomic potential of the triterpenes from the skull sub-samples NH2968 and NH2969 was investigated^35^. *L. orientalis* could be easily excluded as a source since the archaeological samples do not contain oleanonic acid, the predominant triterpene of *L. orientalis* according to our analysis of the fresh resin sample and the literature^36^. On the other hand, in addition to oleanolic acid (VI, Fig. 4) - a triterpene commonly found in plants^37^ -, a series of unusual triterpenoids was identified based on their mass spectra in electron impact, supplemented by field-ionization for molecular weight determination. This compound series occurs in substantial relative amounts in sample NH2968 as well as in a fresh resin sample collected from a *S. officinalis* tree grown in Israel (Fig. 5). They correspond to 6β-hydroxy- or 6-keto- derivatives of oleanolic acid (VII-IX, Fig. 4, mass spectra in Fig. S10) previously reported to occur as minor components of the Asian styrax species *Styrax tonkinensis*^38^. The close similarity of the triterpenic composition of *S. officinalis* resin and the skull sample NH2968, supplemented by the presence, in the latter, of lignan compounds (IV and V, Fig. 4) typically found in the bark of *S. officinalis*^39^, lead us to identify the resinic constituent of the skull sample as an exudate from *S. officinalis*. Finally, the late eluting compounds in the archaeological sample (X–XIV, Fig. 4), absent from the fresh resin sample (Fig. 5), were identified as epoxylactone triterpenoids structurally related to triterpenoids (VI-IX, mass spectra in Fig. S11)^38^ and representing their alteration products (similar compounds have notably been observed after oxidative experiments on oleanolic and ursolic acids using irradiation or chemical reactants)^40^,^41^. This was confirmed by means of a laboratory experiment using fresh resin of *S. officinalis* under radical oxidative conditions, compounds VI to IX being affected by autoxidation processes and leading to compounds X-XIV (Fig. 5).

## Discussion

### Preservation

The cave (about 8 × 4 m) is located on the eastern, drier slope of a cliff on the right bank of the Nahal Hemar and has a narrow entrance (about 1.0 × 0.7 m), guaranteeing relatively stable environmental conditions^12^ (a mean annual temperature of 20 °C and an average precipitation of 100 mm)^42^. The organic compounds found on the archaeological artefacts have benefitted from undergoing relatively limited degradation in the cave context as compared to open-air sites in the region, known for poor biomolecular preservation^43^. The identification of small proteins such as SLRPs is indeed quite remarkable, as these proteins have not been reported in studies of animal binders^44^,^45^, or in the recent characterisation of collagen adhesives from archaeological sites with good organic preservation: in Switzerland (Neolithic waterlogged site in Zurich)^46^, and in China (Bronze Age Xiaohe Cemetery, Taklamakan Desert)^47^. They were however identified in 43 and 700 ka old animal bones found in permafrost^48^,^49^. The exceptional preservation of the proteins from the collagen flake in particular (with high coverages of the collagen proteins) gives a proxy for the potential for preservation of the organics directly related to artefacts recovered from within the cave. However, as the flake itself has been radiocarbon dated as at least a thousand years younger than the layer in which the artefacts were found, it gives little clue as to the manufacture of the collagen coating. It is plausible that the gelatinous flake might simply be a natural product of diagenesis of skin and/or bone (a rare phenomenon but observed in cave sites elsewhere in the Mediterranean)^50^, rather than remnants left by the PPNB visitors of the cave producing the coating substance for their artefacts. Indeed, the original archaeological report of the cave mentions that “a sticky non-greasy, water-soluble substance was found to exude from many of the mammal bones. This could be concentrated urine, or some kind of dung extract. The material also had a pungent odour”^51^.

The presence of collagen, while attested in all samples by their amino acid composition (especially Gly, Pro, Hyp), was confirmed by proteomics only in the samples that presented a high concentration of amino acids. Interestingly, in the cases where the amino acid concentration was low, it was shown that the collagen was mixed with a plant product. It is possible that the addition of these plant-based products, particularly the resin, might have hampered collagen extraction (resulting in low collagen concentration as detected by mass spectrometric analysis) or alternatively contributed to the breakdown of the collagen (as indicated by the absence of diketopyrrole and diketopiperazine derivatives), perhaps as a result of processing the material. In addition, the recovered organic matter would have undergone some degradation upon ageing, as indicated by the presence of oxidized derivatives of triterpenoids. Such alteration processes could explain the presence of numerous unidentified cinnamate and benzoate derivatives (base peak fragments at *m*/*z* 131 and 105) in the archaeological sample (Fig. 4), which may be formed by coupling reactions between benzoic and cinnamic acids and other constituents of the organic substance.

### Archaeological significance

The baskets in Nahal Hemar were made in the close twining technique with twisted strands of plants (reeds, rushes or grasses)^52^ and were coated on the inner and outer surfaces, most likely for lining/waterproofing the baskets. But as only fragments remain, such as in basket #448, it is not known which sub-sample corresponds to the inner or the outer surface. Despite this, both sub-samples show very different results, with one (NH2826) yielding matches to bovine collagen and little else, whereas another (NH2825) yielded remarkably high amounts of a sea squill protein. This is the earliest find of a protein of botanical origin identified in archaeological organic residues (cereal proteins have been identified in a 2500-year old bread from China)^53^; the identification of this sea squill protein may have been facilitated by the natural abundance of charybdin, the main protein constituent in the large bulb of this particular plant^29^ (see SI). The distribution of amino acids in charybdin (Fig. S12) is characterised by a low percentage of glycine, indicating that about twice as much charybdin is necessary to bring the concentration of glycine to its 19% value in NH2825 (compared to 45% in NH2826). Such a difference either indicates that the plant was intentionally mixed with the collagen in NH2825 (which would also be in agreement with the presence of plant triterpenoids and phytosteroids in the lipid extract, as well as plant pyrolysis products) or that a substantial amount of contamination was introduced during the use of the artefact (such as leaching during storage and/or transport of the sea squill plant in the basket). Sea squill (also called medicinal squill) has been known since antiquity and its bulb used for its pharmaceutical properties such as diuretic and cardiotonic^29^. Considering its importance, it is possible that the plant was used as early as the Neolithic and took part in the ritual practices carried out within the cave.

Likewise, the choice of collagen as raw material for the coating, considering the presence of natural asphalts between the cave (now inaccessible from ground level) and the valley, as well as the widespread use of bitumen during the Neolithic period, is intriguing. At the time of the cave occupation, the wadi bottom was completely filled with sediments (as confirmed by the discovery of hyena remains in the superficial archaeological layers)^54^ which allowed access to the cave but is likely to have covered the oil seep so it is plausible that people might not have been aware of its presence^16^. Another possibility is that the objects were imported to the cave, perhaps over long distances, by people who did not use bitumen^54^. Without other evidence of similar manufacturing, it is however difficult to determine if this represents an established practice or a single event. In the basket at least, the collagen was unambiguously identified as bovine. Placed in the Neolithic context, this finding is significant considering the importance that cattle had in this period, whether for ritual (wild cattle burial)^55^ or for secondary products^56^. The cattle bones found in Nahal Hemar are large and either from aurochs or from a large domestic cattle^51^: as no cut marks were found, they might have been placed there for ritual purposes as well.

The application of the styrax resin on the skulls is more comprehensible in the cultural context. This fragrant resin could have been used to perfume these ritual objects, considered by some archaeologists as artefacts for an ancestor cult, and by others as the seat of the life-force honoured by people to ensure fecundity and well-being^10^. The first mention of styrax resin in ancient texts is dated from the 5^th^ c. BC in the travel writings of Herodotus^57^, about 7500 years after the occupation of the Nahal Hemar cave. Scientific evidence of the use of styrax in an archaeological context is extremely rare in the literature and the oldest evidence^1^, to the best of our knowledge, corresponds to a resin (analytical results not detailed in the paper) collected in Egyptian vestiges from the XII^th^ and XIII^th^ dynasty. This conclusion was made by comparison with a commercial resin, but based on the present work, it appears that all the resins commercially available under the label “*Styrax officinalis*” that we have investigated turned out to be mixtures of substances of other origins. This can be explained by the difficulty of obtaining resin from *S. officinalis*, which is only produced under specific conditions (suitable climate, in response to an intentional incision or injury of the bark) and with an extremely low yield^32^. Concerning our own sampling of fresh resin, it took a long period of time to collect sufficient amounts (a few milligrams) of resin for analytical purposes, and pure resin could not be obtained (resin was mixed with bark). It seems likely that Neolithic people produced the resin in the same manner as we did, which explains the presence of lignans IV and V (Fig. 4)- typical constituents of the bark from *S. officinalis*^39^- both in the archaeological and in the fresh resin samples. In any case, the identification of *S. officinalis* resin within the ingredients of the decorative organic coating of the skull corresponds to the earliest evidence of the use of an odoriferous resin in a cultural context.

## Conclusions

The new analytical evidence obtained in this study has highlighted the diversity of materials comprising the exceptionally well-preserved organic coatings from artefacts of the Nahal Hemar cave, more than 10,000 years old. The complexity and originality of these coatings, in particular the addition of an odoriferous resin only on the skulls (possibly due to its rarity and efforts required to obtain sufficient quantity), is unique and the earliest known evidence of the use of these animal and plant products in the Near East. It represents a rare insight into the capacity of Neolithic people who already domesticated various cereals, chickpeas, broad bean, as well as goat, sheep, cattle and pigs that allowed for stable and flourishing economy that drove the social systems into initial hierarchies. Domestic building constructed from stone and mudbricks saw also the investments in carving tall, incised T-shaped limestone pillars for the construction of the ceremonial center of Göbekli Tepe. In this dynamic Levantine society technological inventions were made for domestic purposes such as digging wells and for scheduled rituals. Flax, already used for making strings, was domesticated, and employed in making textiles by twining. In the Neolithic villages extensive use of lime plaster was practiced for the domestic and holy buildings as well as shaping human statues, and modelling skulls and in the rare case described above, with collagen. The presence of artisans is attested in the lapidary technique for beads’ production and carving of stone masks. Several of the earliest Neolithic inventions demonstrating the experimental efforts needed to optimise resources for practical (strengthening baskets) and cultural purposes (decorated skulls) were uncovered in the dry Nahal Hemar cave in the Judean desert, Israel.

## Methods

All archaeological samples are sub-samples from the materials analysed in the original 1995 study^18^, originally obtained from the Israel Museum. Fresh resins of *L. orientalis* and *S. officinalis* were used for comparative GC/MS analyses: the first was obtained from ROBERTET Company and the second, not commercially available, was collected directly on a tree certified as *S. officinalis* and located at Yodfat in Israel (32°50′13.25″ N, 35°16′17.19”E).

### Pyrolysis gas-chromatography mass spectrometry (py-GC/MS) analysis

All samples were analysed by normal flash pyrolysis GC/MS. The samples were pyrolysed using a CDS (Chemical Data Systems) 5200 series pyroprobe pyrolysis unit by heating at 600 °C for 20 s to fragment macromolecular components. These fragments were then analysed using an Agilent 7890A gas chromatograph fitted with a HP-5 fused column (J&W Scientific; 5% diphenyl-dimethylpolysiloxane; 30 m, 0.32 mm i.d., 0.25 μm film thickness) coupled to an Agilent 5975C MSD single quadrupole mass spectrometer operated in electron ionization (EI) mode (scanning a range of *m*/*z* 50–650 at 1 scan s^−1^ with a 1 minute solvent delay; ionization energy 70 eV). The pyrolysis transfer line and injector port temperatures were set at 350 °C, the heated interface at 280 °C, the EI source at 230 °C and the MS quadrupole at 150 °C. Helium was used as the carrier gas and the samples were introduced in split mode in a ratio of 5:1. The oven was programmed from 40 °C (held for 5 minutes) to 250 °C at 4 °C min^−1^, then to 320 °C at 20 °C min^−1^, were it was kept for 5 min. Compounds were identified by comparison with spectra from the literature.

### Gas-chromatography mass spectrometry (GC/MS)

The lipid material from the archaeological samples (20–500 mg) was extracted by sonication using a mixture of dichloromethane (DCM)/methanol (MeOH) (1:1, v/v) by adaptation of an established protocol^58^. After filtration through celite, the solvents were removed under reduced pressure to obtain the total lipid extract (TLE). An aliquot of the TLE, dissolved in DCM, was acetylated using 1 mL of acetic anhydride and 20 μL of N-methylimidazole (catalyst) at ambient temperature for 20 min. After addition of MeOH followed by removal of the residual reactants and solvent under reduced pressure, the acetylated extract was esterified with diazomethane in diethyl ether (1 mL) at ambient temperature for 30 min. The excess of diazomethane was removed under a nitrogen flux and the derivatized TLE was then fractionated on a silica gel column into an apolar fraction (elution with DCM/ethyl acetate (EtOAc), 8:2 v/v) analysed by GC/MS and GC/HRMS, and a more polar fraction (elution with DCM/MeOH (1:1 v/v) not further investigated. Sample material remaining after lipid extraction (the “residue”) was subjected to acid methanolysis with CH_3_OH/HCl (6N, 110 °C, 7 to 9 h). After liquid-liquid extraction, the aqueous phase was recovered and the released amino acids were derivatized first using BuOH/HCl (3N, 12 mL, 100 °C, 1 h) and secondly, after evaporation under reduced pressure, with trifluoroacetic anhydride (3 mL, ambient temperature, 3 h). The residual reactants were removed by evaporation to dryness under N_2_. Before GC/MS injection, separation of the constituents was performed on a silica gel column following the protocol described in literature^58^. To perform the oxidation experiment on a fresh sample of *S. officinalis* resin, acetonitrile (ACN, 5 mL), *tert*-butyl-hydroperoxide (5,5 M in decane, 500 μL) and azobisisobutyronitrile (15 mg) were added in this order to about 10 mg of TLE of the resin and the reaction was operated at 50 °C for 13 h under an air atmosphere. Following liquid-liquid extraction, the organic phase was collected and treated as described previously prior to GC/MS analysis. GC/MS analyses were performed on a Thermo Scientific GC Trace Ultra gas chromatograph equipped with a programmed temperature vaporizing injector and a HP5 MS column (30 m, 0.25 mm i.d., 0.25 μm film thickness) connected to a triple quadripole Thermo Scientific TSQ Quantum mass spectrometer using helium as carrier gas and the following temperature program: 70 °C (held for 1 min) to 200 °C at 10 °C min^−1^, then to 320 °C at 4 °C min^−1^ followed by an isothermal period of 40 min. The transfer line temperature was set at 320 °C. MS spectra were collected in electron impact (ionization voltage of 70 eV) or in chemical ionisation mode (isobutane as gas reactant gas) and registered in full-scan mode (scan from 50 to 800 *m*/*z*).

GC/MS analyses in the field ionization mode (FI) were carried out on a Agilent Technologies 7890A gas chromatograph coupled to a JEOL Accu TOF GCV JMS-T100GCV mass spectrometer using a scan from 35 to 800 *m*/*z*. The instrument was equipped with a split/splitless injector used in a splitless mode and a DB5UI column (20 m, 0.18 mm i.d., 0.18 μm film thickness) using helium as carrier gas and the following temperature program: 70 °C (held for 1 min) to 200 °C at 10 °C min^−1^, then to 320 °C at 4 °C min^−1^ followed by an isothermal period of 10 min.

### Amino acid analysis

In total 28 amino acid analyses were undertaken on:

1 subsample of NH2824 (NEaar 7742; NH2824O-3), analysed in triplicate.

2 subsamples of basket sample 448-1208-210 NH2825 (NEaar 9127–9128; NH2825.1–2), each analysed singly or in duplicate.

3 subsamples of basket sample 448-1208-210 NH2826 (NEaar 9997–9999; NH2826.1–3), each analysed at least in duplicate.

3 subsamples of NH2968 (NEaar 10000–2; NH2968.1–3), each analysed singly or in duplicate.

3 subsamples of NH2969 (NEaar 10003–5; NH2969.1–3), each analysed in duplicate.

Samples were prepared using modified procedures of Penkman and Penkman *et al.*^59^,^60^. NH2824 was bleached before analysis, involving treatment with 12% NaOCl for 15 seconds, the bleach removed by rinsing with water and methanol, and then dried overnight in the fume hood. All samples were treated with 7 M HCl under N_2_ at 110 °C for 24 h (H*), referred to as the ‘total hydrolysable’ amino acid’ fraction (THAA), to release the peptide-bound amino acids, thus yielding the ‘total’ amino acid concentration. Samples were then dried by centrifugal evaporator and rehydrated for reversed phase high pressure liquid chromatography (RP-HPLC) analysis with 0.01 mM L-homo-arginine as an internal standard. The primary amino acid compositions of the samples were analysed in duplicate by an Agilent 1100 HPLC using fluorescence detection following a modified method of Kaufman and Manley^61^. During preparative hydrolysis both asparagine and glutamine undergo rapid irreversible deamidation to aspartic acid and glutamic acid respectively^62^. It is therefore not possible to distinguish between the acidic amino acids and their derivatives and they are reported together as Asx and Glx.

### Proteomic analysis

The sample was resuspended in 200 μL 50 mM ammonium bicarbonate, heated for 3 hours at 65 ^o^C and digested with 1 μL of 1 μg μL^−1^ trypsin. The solution was then acidified to 0.1% trifluoroacetic acid (TFA), sample clean-up using a C_18_ pipette tip was carried out into 50% acetonitrile (ACN) and the solution of eluted peptides concentrated by evaporation and resuspension in 10 μL 0.1% TFA. 1 μL of sample was then analysed by LC-MS/MS (Waters nanoAcquity UPLC coupled to a Thermo Scientific LTQ Velos Dual Pressure Linear Ion Trap mass spectrometer) on which the peptides were concentrated on a pre-column (20 mm x 180 μm) then separated on a 1.7 μM Waters nanoAcquity BEH (Ethylene Bridged Hybrid) C_18_ analytical column (75 mm × 250 μm i.d.), using a gradient from 99% buffer A (0.1% formic acid (FA) in H_2_O)/1% buffer B (0.1% FA in ACN) to 25% B in 45 min at 200 nL min^−1^. Peptides were selected for fragmentation automatically by data dependent analysis. Proteomics data files were searched using Mascot v2.2.06 (Matrix Science) against the publicly available UniProt (Universal Protein Resource) database (www.uniprot.org). Standard searches were carried out using two missed cleavages, error tolerances of 0.5 *m*/*z* units (MS and MS/MS) and variable oxidation of methionine and hydroxylation of proline and lysine and deamidation of asparagines and glutamine modifications. Raw files have been made available on PeptideAtlas (http://www.peptideatlas.org/PASS/PASS00915).

## Additional Information

**How to cite this article**: Solazzo, C. *et al.* Identification of the earliest collagen- and plant-based coatings from Neolithic artefacts (Nahal Hemar cave, Israel). *Sci. Rep.* **6**, 31053; doi: 10.1038/srep31053 (2016).

## Supplementary Material

Supplementary Information

## Figures and Tables

**Figure 1:**
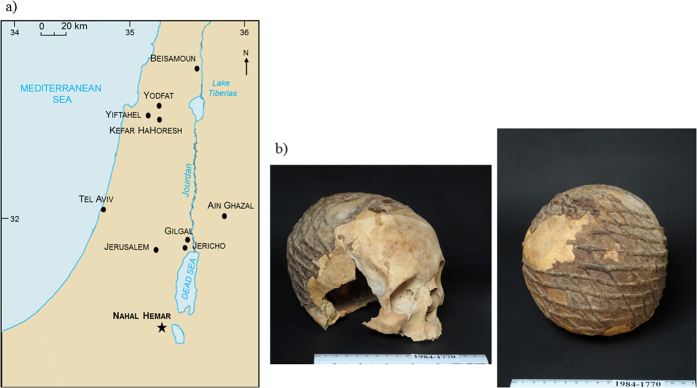
(**a**) Location of the Nahal Hemar cave, 17 kms west of the Dead Sea and of other Israeli PPNB sites mentioned in the article (map modified from https://www.cia.gov/library/publications/the-world-factbook/geos/is.html, The World Factbook, Washington, DC: Central Intelligence Agency, 2016), (**b**) Human skull #515 with a net pattern decoration from which sub-samples NH2968 and NH2969 have been collected (accession number IAA No. 1984–1770, Photo Clara Amit, Courtesy of the Israel Antiquities Authority).

**Table 1:** Dates from Nahal Hemar obtained after the excavations in 1983 (calibrated by Calpal on line 2007, http://www.calpal-online.de).

Layer	Material	Lab Number	^14^C BP	Cal BC
1	cloth	OxA-1013	660 ± 200	1296 ± 168 A.D.
3A	charcoal	RT- 650	8100 ± 100	7061 ± 185
3A	charcoal	Pta-3650	8270 ± 80	7312 ± 122
3A	charcoal	BM-2298	8250 ± 70	7312 ± 122
3B	Knots of net	OxA-1014	8600 ± 120	7708 ± 146
3B	Twined napkin	OxA-1015	8500 ± 220	7975 ± 266
4	Linen yarn.1	Pta-3625	8850 ± 90	7986 ± 174
4	Linen yarn.2	BM-2299	9120 ± 300	8335 ± 422
4	Knotted fabric	OxA-1016	8810 ± 120	7940 ± 203

**Table 2:** Sample information and main results.

Artefacts^a^	Archaeological reference^a^ and description	Sub-sample numbers^b^	Position	Period^c^	Composition
Organic Flake #672	“Resin”: yellow lump, resin-like aspect	NH2824	Layer 3	7300–7050 cal. BC	Collagen
Basket #448	1208–210 B: Black lump with vegetal debris on surface	NH2825	Layer 4	—	Collagen, squill
	1208–210 C: Brown lump with vegetal imprint	NH2826			Collagen
Skull #515	Skull: Black lump of the basis	NH2968	Layer 4	8920–8430 cal. BC	Collagen, styrax
	Skull: Black lump of the net pattern	NH2969			Collagen, styrax

^a^Accession numbers as given in Connan *et al.*^18^.

^b^Laboratory references.

^c^Radiocarbon dates obtained at the Weizmann Institute.

**Table 3:** Proteomic results in sample NH2824 and basket samples NH2825/26: proteins matched with total number (#) of non-redundant peptides with score >40; SLRP stands for Small-Leucine-Rich Proteins.

	Collagen	#	Blood	#	SLRPs	#	Charybdin	#	Others	#
Flake NH2824	α-1(I), α-2(I), α-1(VI), α-2(VI), α-3(VI)	138	Hemoglobin, Fibrinogen, Complement C3^a^	26	Decorin, Lumican, Mimecan	9	—	—	—	—
Basket NH2825	—	—	—	—	—	—	Ribosome-inactivating protein	6	—	—
Basket NH2826	α-1(I), α-2(I), α-3(VI)	29	a	—	Fibromodulin, Biglycan, Aggrecan core protein	7	—	—	Thrombospondin-1, Keratin cytoskeletal 1	7

^a^Serum albumin identified (although the identification of serum albumin may be genuine, it is ignored here as it is likely to be a column carry-over contaminant due to its generic use in proteomic laboratories as a standard, confirmed with it being present in our blank analyses). Details in SI.
