# Supplementary material for: Identification of the earliest collagen- and plant-based coatings from Neolithic artefacts (Nahal Hemar cave, Israel)
Source: Sci Rep. 2016 Aug 9;6:31053. doi: 10.1038/srep31053 (PMC4977467; doi:10.1038/srep31053)
Supplement: Supplementary Information [file srep31053-s1.pdf]

SUPPLEMENTARY INFORMATION

Identification of the earliest collagen- and plant-based coatings from Neolithic artefacts (Nahal Hemar cave, Israel)

Caroline Solazzo[1], Blandine Courel[2], Jacques Connan[3], Bart E. van Dongen[4], Holly Barden[4], Kirsty Penkman[5], Sheila Taylor[5], Beatrice Demarchi[5], Pierre Adam[2], Philippe Schaeffer[2], Arie Nissenbaum[6], Ofer Bar-Yosef[7], Mike Buckley[8]

[1] Museum Conservation Institute, Smithsonian Institution, Suitland, MD 20746

[2] Laboratoire de Biogéochimie Moléculaire, Institut de Chimie de Strasbourg, UMR 7177, Université de Strasbourg, 67200 Strasbourg, France

[3] 23 rue Saint-Exupéry, 64000 Pau, France

[4] School of Earth, Atmospheric and Environmental Sciences, Williamson Research Centre for Molecular Environmental Science, University of Manchester, Manchester, M13 9PL, United Kingdom

[5] BioArCh, Department of Chemistry, University of York, York, YO10 5DD, United Kingdom

[6] Department of Earth and Planetary Sciences, Weizmann Institute of Science, Rehovot 76100, Israel

[7] Department of Anthropology, Harvard University, Cambridge, MA 02138, USA

[8] Faculty of Life Sciences, Manchester Institute of Biotechnology, University of Manchester, Manchester, M1 7DN, United Kingdom

<u>Corresponding authors:</u>

Solazzo Caroline, solazzo.c@gmail.com,
Museum Conservation Institute, Smithsonian Institution, Suitland, MD 20746

Buckley, Mike, m.buckley@manchester.ac.uk
Manchester Institute of Biotechnology, The University of Manchester, Manchester, M1 7DN, United Kingdom

The Nahal Hemar cave

The cave was first discovered in the 1950s and 60s by Bedouins who were searching for Dead Sea scrolls; they dug along the entrance of the cave but found nothing[1]. Later the cave was rediscovered and excavated by archaeologists in 1983[2]. A test excavation in March along the Western wall, opposite the entrance, revealed the modelled skulls and other bone and stone artefacts. This was followed by a systematic excavation in June by Ofer Bar-Yosef and David Alon which established the stratigraphy along the Northern wall. The cave is small (about 4 x 8 m), and was covered with large boulders that had fallen from the ceiling. Neolithic finds were located under the boulders, so that these rocks must have fallen after the Neolithic occupation of the cave. Between the first survey and the excavation of the cave, the central part had been plundered but original deposits were found under the boulders. The stratigraphy were described as follow in Bar-Yosef, 1985[1] and 1988[2]:

Stratum 1: about half a meter thick; contained Early Bronze I and Byzantine-Early Arabic sherds, as well as "coprolites, broken twigs, and gravel formed by the disintegration of a rock layer in the cave wall".

Stratum 2: 0.35-0.45 m thick; contains few Neolithic finds, gravel and coprolites. "The gravel was due to the crumbling of the rock caused by the use of the cave as a sheep-pen"

Stratum 3 (3A and 3B): 0.60 m thick; rich in Neolithic finds (such as threads, flint tools, animal bones), including the remains of a hearth (layer 3A, near the North wall) from which charcoal were used for radiocarbon dating (Table 1A).

Stratum 4: depth varies as it covered the uneven cave floor and material between the stone boulders; it contained stalagmites, white sand and coprolites. The layer produced the important artefacts such as the skulls, a sickle and the head gear. All deposits were sieved, and objects from the dumps were found to complete some of the excavated artefacts.

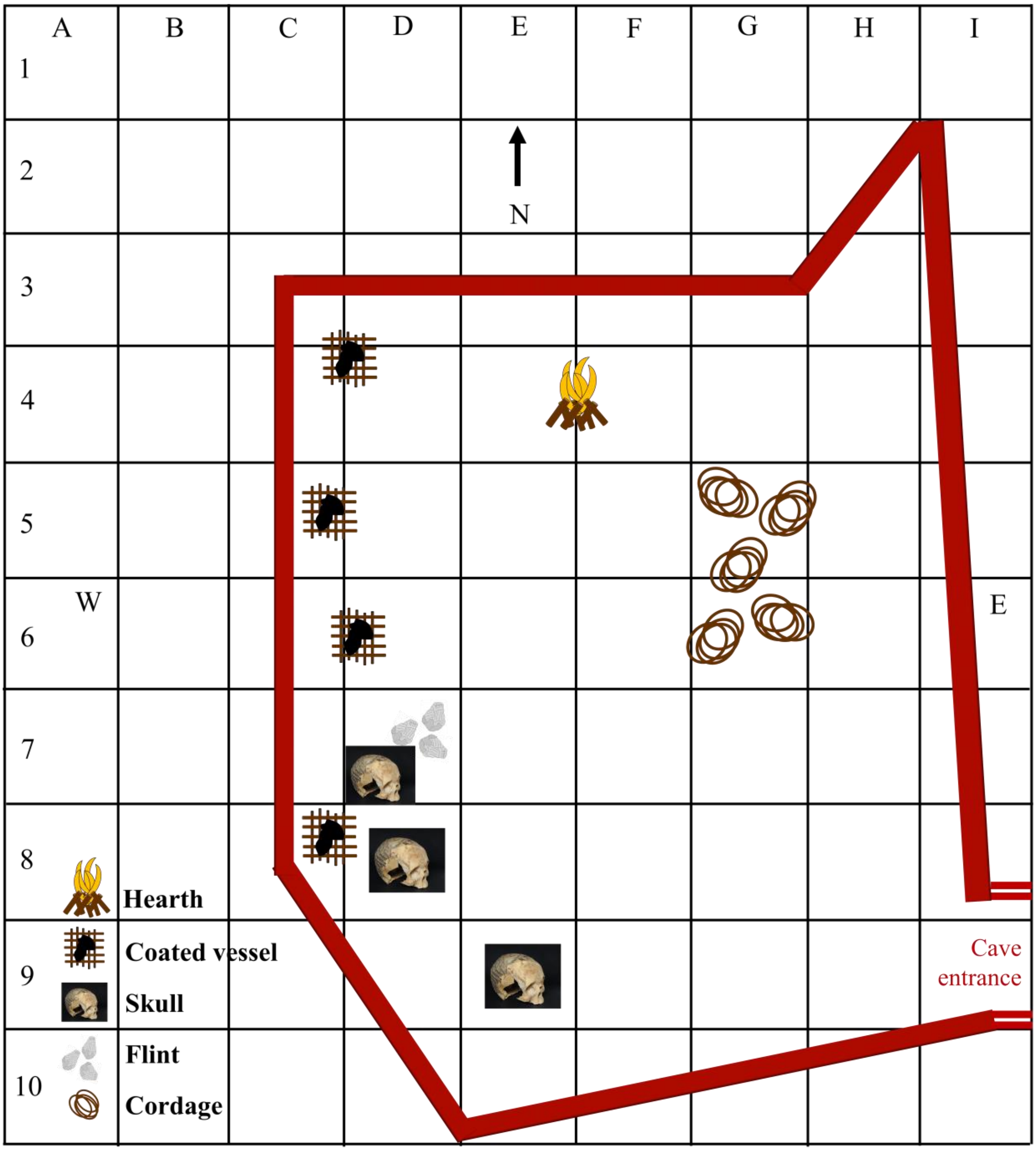

Outline of the cave, showing the approximate positions of finds and the skulls near the Western wall (one square represents 1 m^2).

Amino acid distribution obtained by RP-HPLC

Collagen is the major protein identified in archaeological bones and skin. It is a fibrous protein found in skin, sinews, and bone and consists of a coil of three chains (two identical chains α1 and one α2), forming a triple helix where each chain is made of repetitive glycine-Xaa-Yaa motifs where Xaa and Yaa can be almost any other amino acid but is mostly proline or hydroxyproline and Yaa respectively. As such, glycine accounts for approximately one third of the amino acid content, giving it a distinctive amino acid composition signature.

Table S1: Distribution of amino acids (with standard deviation in brackets) in the archaeological samples, compared to a bone reference; n refers to the number of analyses and the average total concentration in amino acids is indicated in µmol/mg

Sample	Flake #672	Basket #448	Basket #448	Skull #515	Skull #515	Reference bone
Sub-sample	NH2824	NH2825	NH2826	NH2968	NH2969	
% Asx	7.15 (0.33)	10.80 (0.38)	5.18 (0.22)	11.12 (0.98)	11.80 (0.37)	6.24
% Glx	9.97 (0.38)	12.95 (0.73)	11.49 (0.69)	10.99 (0.56)	12.53 (0.60)	9.74
% Ser	5.14 (0.24)	7.86 (1.64)	1.87 (0.13)	5.91 (1.58)	7.12 (0.65)	3.88
% Thr	3.52 (0.30)	4.55 (2.81)	2.55 (0.08)	4.52 (0.55)	5.83 (0.13)	2.42
% His	0.72 (0.04)	0.88 (0.06)	0.57 (0.05)	0.20 (0.45)	0.38 (0.59)	1.08
% **Gly**	**43.25 (1.12)**	**18.95 (2.68)**	**45.32 (1.85)**	**34.09 (2.14)**	**18.96 (1.09)**	**45.43**
% Arg	4.74 (0.08)	2.30 (0.16)	5.00 (0.30)	2.86 (0.41)	1.61 (0.80)	5.74
% Ala	10.45 (0.17)	10.15 (0.77)	11.70 (0.21)	9.67 (0.86)	10.17 (0.38)	13.63
% Tyr	0.24 (0.07)	2.01 (0.11)	0.40 (0.12)	0.49 (0.45)	1.30 (0.65)	0.52
% Val	4.87 (0.17)	6.77 (0.86)	4.18 (0.20)	5.60 (0.09)	7.80 (0.36)	3.09
% Phe	2.22 (0.26)	5.27 (1.11)	2.43 (0.08)	4.63 (1.57)	6.39 (0.48)	1.83
% Leu	5.33 (0.82)	11.32 (1.32)	6.94 (0.25)	5.82 (2.09)	9.08 (0.40)	4.75
% Ile	2.39 (0.44)	6.19 (0.63)	2.37 (0.10)	4.09 (0.22)	7.03 (0.32)	1.64
n	3	4	8	5	6	1
Conc. µmol/mg	27.34	0.15	1.82	0.18	0.11	2.33

NH2824: Amino acid profile obtained by gas chromatography

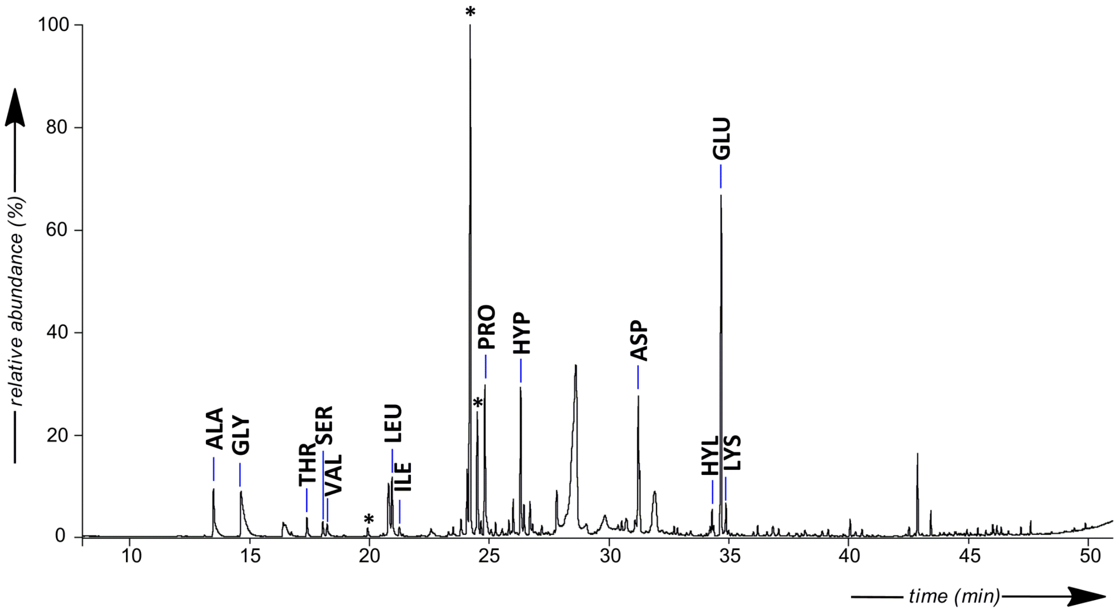

Figure S1: Partial gas chromatogram of the hydrolysed residue (HCl/CH_3OH, 6N, 7h) remaining after solvent extraction of the organic material **NH2824**. (ALA) alanine, (GLY) glycine, (THR) threonine, (SER) serine, (VAL) valine, (LEU) leucine, (ILE) isoleucine, (PRO) proline, (HYP) hydroxyproline, (ASP) aspartic acid, (HYL) 5-hydroxylysine, (GLU) glutamic acid, (LYS) lysine. Acids are identified as their butyl ester derivatives and alcohol and amine functions are characterised as their TFAA derivatives. Amino acids have been identified based on their mass spectra in electron impact and chemical ionization and by comparison with the NIST database. * = Carbohydrates.

NH2825: Amino acid profile obtained by gas chromatography

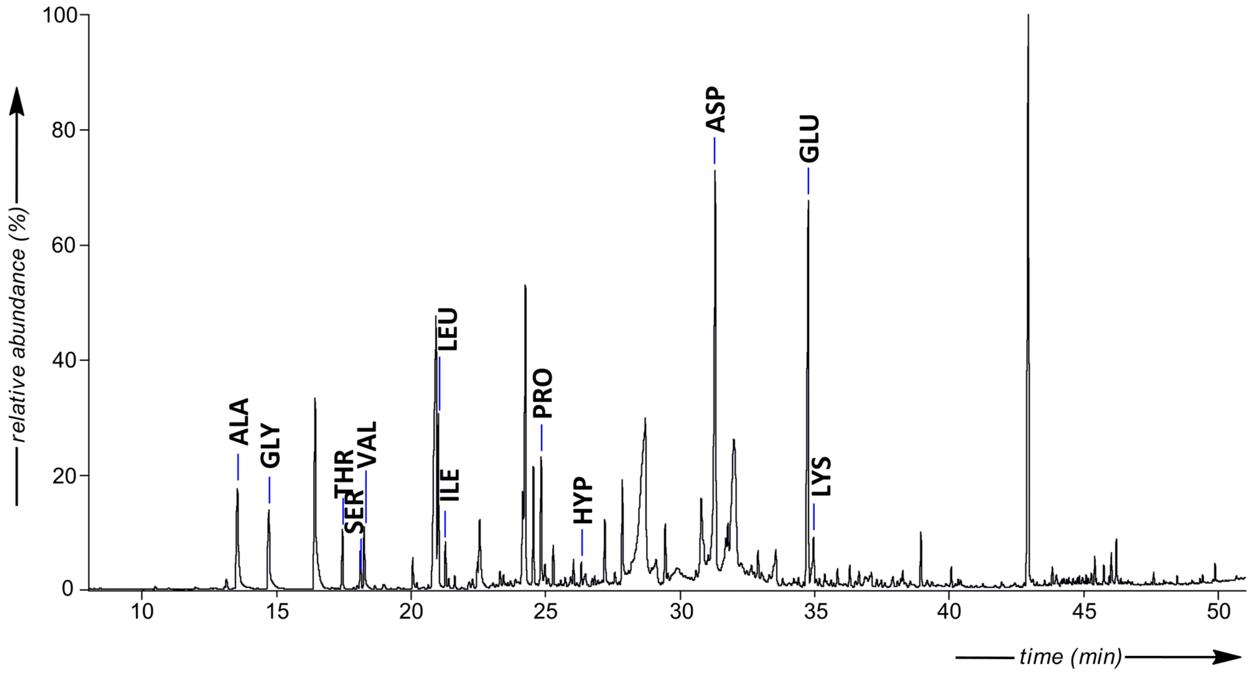

Figure S2: Partial gas chromatogram of the hydrolysed residue remaining after solvent extraction (HCl/CH_3OH, 6N, 9 h) of the basket sample **NH2825**. (ALA) alanine, (GLY) glycine, (THR) threonine, (SER) serine, (VAL) valine, (LEU) leucine, (ILE) isoleucine, (PRO) proline, (HYP) hydroxyproline, (ASP) aspartic acid, (GLU) glutamic acid, (LYS) lysine. Acids are identified as their butyl ester derivatives and alcohol and amine functions are characterised as their TFAA derivatives. Amino acids have been identified based on their mass spectra in electron impact and chemical ionization and by comparison with the NIST database.

NH2968: Amino acid profile obtained by gas chromatography

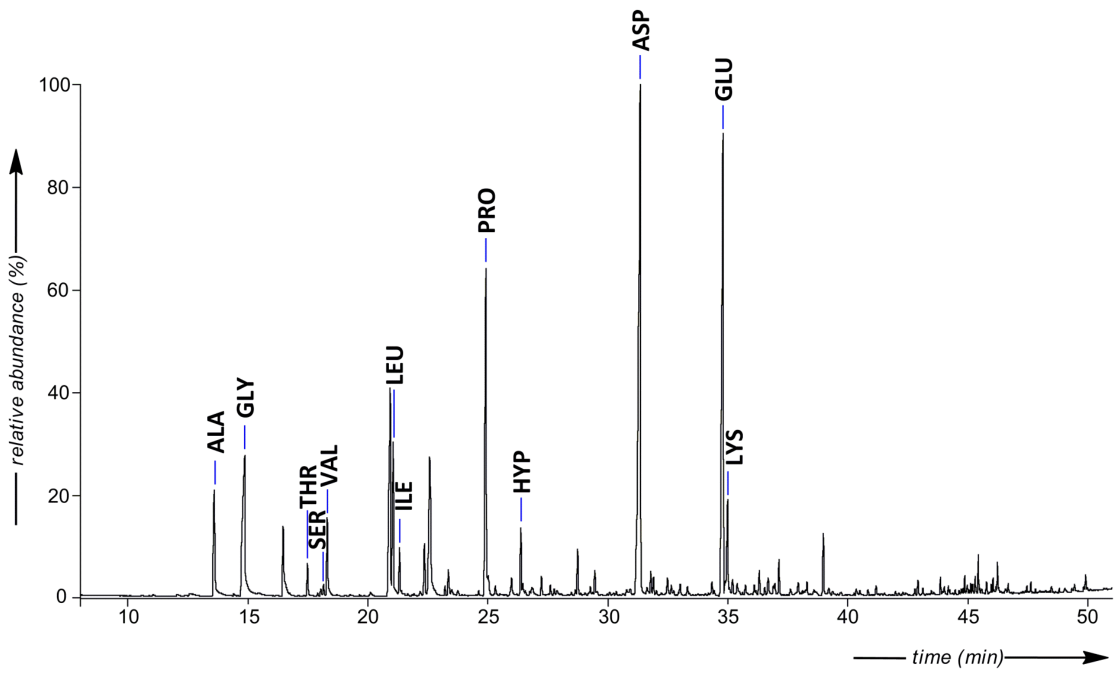

Figure S3: Partial gas chromatogram of the hydrolysed residue (HCl/CH_3OH, 6N, 9h) remaining after solvent extraction of the skull sample **NH2968**. (ALA) alanine, (GLY) glycine, (THR) threonine, (SER) serine, (VAL) valine, (LEU) leucine, (ILE) isoleucine, (PRO) proline, (HYP) hydroxyproline, (ASP) aspartic acid, (GLU) glutamic acid, (LYS) lysine. Acids are identified as their butyl ester derivatives and alcohol and amine functions are characterised as their TFAA derivatives. Amino acids have been identified based on their mass spectra in electron impact and chemical ionization and by comparison with the NIST database.

Identification of pyrolysis products obtained by py-GC/MS

Table S2: Main pyrolysis moieties identified with their possible biological origin as characterised in [3-9]

Peak	Characteristic ions (m/z)	Compound name	Origin
1	53, 66, 68(M)	2-Pentyne	
2	53, 81, 82(M)	2-Methylfuran	
3	77, 78(M)	Benzene	
4	68, 69(M)	Pyrroline	Pro
5	51, 53, 81, 95, 96(M)	2, 5-dimethylfuran	
6	53, 80, 81(M)	1-methyl-pyrrole	Pro, Hyp
7	50, 51, 52, 79(M)	Pyridine	
8	91, 92(M)	Toluene	Phe
9	95, 110(M)	2-ethyl-5-methylfuran	
10	53, 67, 80, 95(M)	1-ethyl-pyrrole	Hyp
11	66, 92, 93(M)	2-methyl-pyridine	
12	53, 80, 81(M)	2-methyl-Pyrrole	Pro, Hyp
13	91, 106(M)	Ethylbenzene	Phe
14	86, 94, 95(M)	2,5-dimethyl-1H-pyrrole	
15	66, 92, 93(M), 94	3-methyl-pyridine	
16	51, 78, 103, 104(M)	Styrene	Phe
17	96, 67, 53	2-methyl-cyclopentenone	
18	79, 106, 107(M)	2-ethyl-pyridine	
19	81, 108(M)	2,5-dimethyl-pyrazine	
20	54, 55, 84(M)	2(5H)-Furanone	
21	80, 94, 95(M)	2,4-dimethyl-pyrrole	
22	79, 106, 107(M)	2,5-dimethyl-pyridine	
23	91, 115, 117, 118(M)	2-propenyl-benzene	
24	91, 120(M)	Propylbenzene	
25	50, 51, 52, 57, 71, 79, 99, 104, 105(M)	2-ethenyl-pyridine	
26	50, 80, 95(M)	2-ethyl-pyrrole	Hyp
27	65, 79, 92, 106, 107(M)	3-ethyl-pyridine	
28	51, 77, 105, 106(M)	Benzaldehyde	Balsam
29	50, 51, 53, 81, 109, 110(M)	5-methyl-2-Furaldehyde	
30	76, 103(M)	Benzonitrile	
31	94, 121, 122(M)	2-ethyl-6-methyl-Pyrazine	
32	66, 94(M)	Phenol	Tyr, Lignin
33	94, 109(M)	2-ethyl-4-methyl-pyrrole	
34	91, 94, 115, 117, 118(M)	beta-Methylstyrene	
35	93, 120, 121(M)	2-ethyl-6-methyl-Pyridine	
36		methylpyrrole-2-carboxylate	
37	67, 94(M)	2-aminopyridine	
38	80, 81, 137(M)	1-pentyl-pyrrole	
39	89, 90, 116, 117(M)	Benzyl nitrile	
40	51, 77, 105, 120(M)	Acetophenone	
41	53, 81, 109, 124(M)	4-methoxyphenol	
42	51, 77, 79, 107, 108(M)	4-methylphenol=p-cresol	Lignin
43	81, 109, 124(M)	2-methoxyphenol=guaiacol	Lignin
44	66, 94, 109(M)	3-acetyl-1-pyrroline	
45	117, 118, 119(M)	Indoline	
46	56, 113(M)	1-methyl-2,5-pyrrolidinedione	

47	56, 107, 108(M)	Ethylpyrazine	
48	89, 90, 116, 117(M)	Benzyl nitrile	
49	54, 69, 97(M)	Pyrrole-2,5-dione=maleimide	Asn
50	84, 99(M)	5-methyl-2-pyrrolidinone	
51	65, 93, 121, 136(M)	Hydroxyacetophenone	
52	77, 107, 121, 122(M)	2,4-dimethylphenol	Tyr
53	77, 107, 121, 122(M)	2,5-dimethyl-phenol	Tyr
54	70, 85, 113(M)	1-acetylpyrrolidine	
55	77, 95, 107, 122, 123, 138(M)	2-methoxy-4-methyl-Phenol=creosol	Lignin
56	107, 122(M)	2,3-dimethyl-phenol	Tyr
57	78, 106, 134(M)	2-Coumaranone	
58	51, 77, 105, 122(M)	Benzoic acid	Balsam
59	91, 104, 164(M)	Methyl hydrocinnamate	Balsam
60	137, 152(M)	4-ethyl-guaiacol	Lignin
61	51, 52, 78, 79, 122(M)	Picolinamide	
62	89, 90, 117(M)	Indole	Trp
63	77, 107, 135, 150(M)	4-vinylguaiacol	Lignin
64	93, 96, 111, 139, 154(M)	2,6-dimethoxy-phenol=Syringol	Lignin
65	77, 103, 131, 161, 162(M)	Methyl cinnamate	Balsam
66	51, 78, 128, 129, 155, 156(M)	Bipyridine	
67	81, 151, 152(M)	Vanillin	Lignin, Balsam
68	50, 154, 155(M)	3-phenyl-pyridine	
69	77, 91, 103, 149, 164(M)	Isoeugenol	Lignin
70	51, 77, 91, 103, 147, 148(M)	Cinnamic acid/Trans Cinnamic acid	Balsam
71	53, 80, 107, 108, 136(M)	3-propyl-phenol	
72	168, 183, 198(M)	1,6-dimethyl-4-(1-methylethyl)-naphtalene	
73	93, 130, 186(M)	Diketodipyrrole	Hyp
74	77, 91, 179, 194(M)	Methoxyeugenol	Lignin
75	70, 97, 125, 168(M)	2,5-diketopiperazine derivative	Pro-Ala
76	55, 57, 60, 73, 129, 143, 185, 228(M)	Tetradecanoic acid	
77	70, 83, 111, 154(M)	Pyrrolidinopiperazine derivative	Pro-Gly, Pro-Lys
78	70, 194(M)	2,5-diketopiperazine derivative	Pro-Pro
79	55, 57, 60, 73, 129, 213, 256(M)	Hexadecanoic acid	
80	55, 57, 60, 73, 129, 227, 171, 185, 270(M)	Heptadecanoic acid	
81	77, 91, 103, 131, 192, 193, 238(M)	Benzyl cinnamate	Balsam
82	55, 57, 60, 73, 129, 185, 241, 284(M)	Octadecanoic acid	
83	103, 115, 117, 131, 219, 264(M)	Cinnamyl cinnamate	Balsam

Profiles obtained by pyrolysis gas chromatography

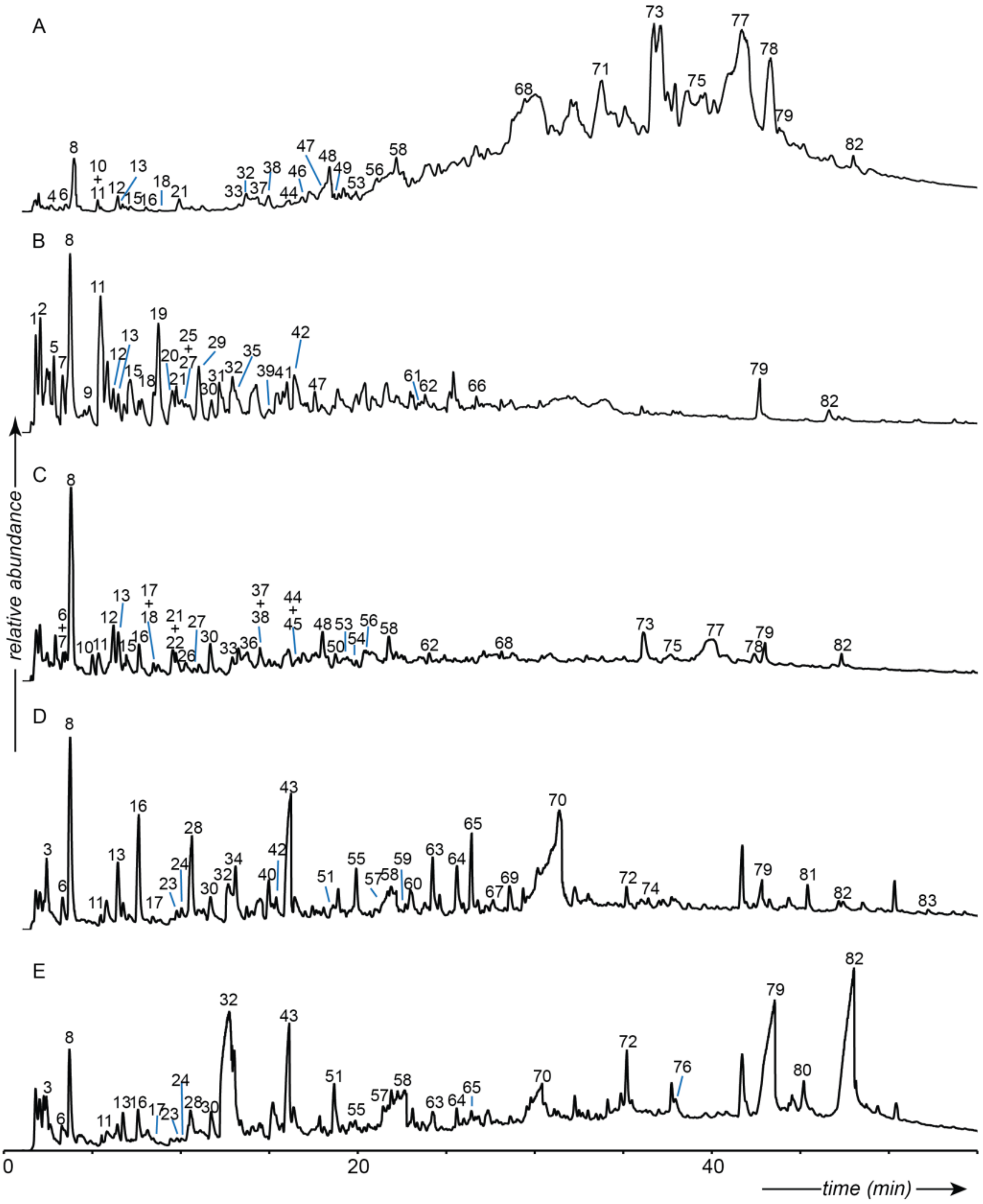

Figure S4: Py-GC/MS total ion current chromatograms of A) NH2824, B) NH2825, C) NH2826, D) NH2868 and E) NH2869. Compound numbers refer to those reported in Table S2.

Identification of proteins by nanoLC-MS/MS

Table S3: Proteomic results on the flake **NH2824**: Protein name, accession number, species, #peptides (#peptides > mascot score 40), percentage coverages, ion score (ion score > mascot score 40) and role of the protein. Proteins were selected if at least three peptides were matched with a score >40.

Protein	Acc. No.	Species	# pep. (>40)	Cov (%)	Ion Score (>40)	Function and location
Collagen alpha-1(I)	CO1A1_BOVIN	*Bos taurus*	540(139)	61	3271(2942)	Structural role Connective tissues
Collagen alpha-2(I)	CO1A2_BOVIN	*Bos taurus*	325(84)	48	2667(2317)	Structural role Connective tissues
Serum albumin	ALBU_BOVIN	*Bos taurus*	47(15)	24	605(520)	Maintain oncotic pressure and transport protein Plasma
Hemoglobin subunit beta	HBB_BOVIN	*Bos taurus*	23(7)	47	210(206)	Oxygen-carrier Blood
Hemoglobin subunit alpha-I/II	HBA_BISBO HBA_BOVIN	*Bison bonasus*	15(4)	44	156(130)	Oxygen-carrier Blood
Collagen alpha-2(VI) chain	CO6A2_HUMAN	*Homo sapiens*	14(5)	12	140(141)	
Decorin	PGS2_BOVIN	*Bos taurus*	23(4)	26	137(121)	Small leucine-rich proteoglycan, involved in collagen fibrinogenesis
Collagen alpha-3(VI) chain	CO6A3_HUMAN	*Homo sapiens*	25(5)	8	119(103)	
Lumican	LUM_BOVIN	*Bos taurus*	18(5)	21	110(107)	Small leucine-rich proteoglycan, involved in collagen fibrinogenesis
Mimecan	MIME_BOVIN	*Bos taurus*	17(3)	17	105(97)	Small leucine-

						rich proteoglycan, involved in collagen fibrinogenesis.
Collagen alpha-1(VI) chain	CO6A1_HUMAN	*Homo sapiens*	11(4)	7	103(89)	
Complement C3	CO3_BOVIN	*Bos taurus*	12(3)	6	90(90)	Protein of the immune system
Fibrinogen gamma-B chain	FIBG_BOVIN	*Bos taurus*	7(3)	12	87(84)	Glycoprotein involved in blood clotting Plasma
Fibrinogen beta chain	FIBB_BOVIN	*Bos taurus*	12(3)	14	85(78)	Glycoprotein involved in blood clotting Plasma
Fibrinogen alpha chain	FIBA_BOVIN	*Bos taurus*	9(4)	9	78(78)	Glycoprotein involved in blood clotting Plasma

Table S4: Proteomic results on the basket samples **NH2825** and **NH2826**: Protein name, accession number, species, #peptides (#peptides > mascot score 40), percentage coverages, ion score (ion score > mascot score 40) and role of the protein. Proteins were selected if at least three peptides were matched with a score >40.

Protein	Acc. No.	Species	# pep. (>40)	Cov. (%)	Ion Score (>40)	Function and location
**NH2825**						
Ribosome-inactivating protein charybdin	RIP_DRIMA	*Drimia maritima*	84(11)	55	335(269)	Protein synthesis inhibitor
**NH2826**						
Collagen alpha-2(I)	CO1A2_BOVIN	*Bos taurus*	116(20)	33	599(544)	Structural role<br>Connective tissues
Collagen alpha-1(I)	CO1A1_BOVIN	*Bos taurus*	165(15)	25	403(337)	Structural role<br>Connective tissues
Serum albumin	ALBU_BOVIN	*Bos taurus*	26(10)	17	314(283)	Maintain oncotic pressure and transport protein<br>Plasma
Collagen alpha-3(VI) chain	CO6A3_HUMAN	*Homo sapiens*	24(4)	5	138(101)	
Fibromodulin	FMOD_BOVIN	*Bos taurus*	26(4)	10	119(112)	Small leucine-rich proteoglycan, involved in collagen fibrinogenesis
Biglycan	PGS1_BOVIN	*Bos taurus*	13(4)	20	115(105)	Small leucine-rich proteoglycan, involved in collagen fibrinogenesis
Keratin, type II cytoskeletal 1	K2C1_HUMAN	*Homo sapiens*	13(4)	18	112(95)	Structural, skin

Aggrecan core protein	PGCA_BOVIN	*Bos taurus*	23(3)	4	94(92)	Small leucine-rich proteoglycan, major component of articular cartilage
Thrombospondin-1	TSP1_BOVIN	*Bos taurus*	5(3)	3	68(63)	Glycoprotein

NH2824: tandem MS spectrum, bovine collagen

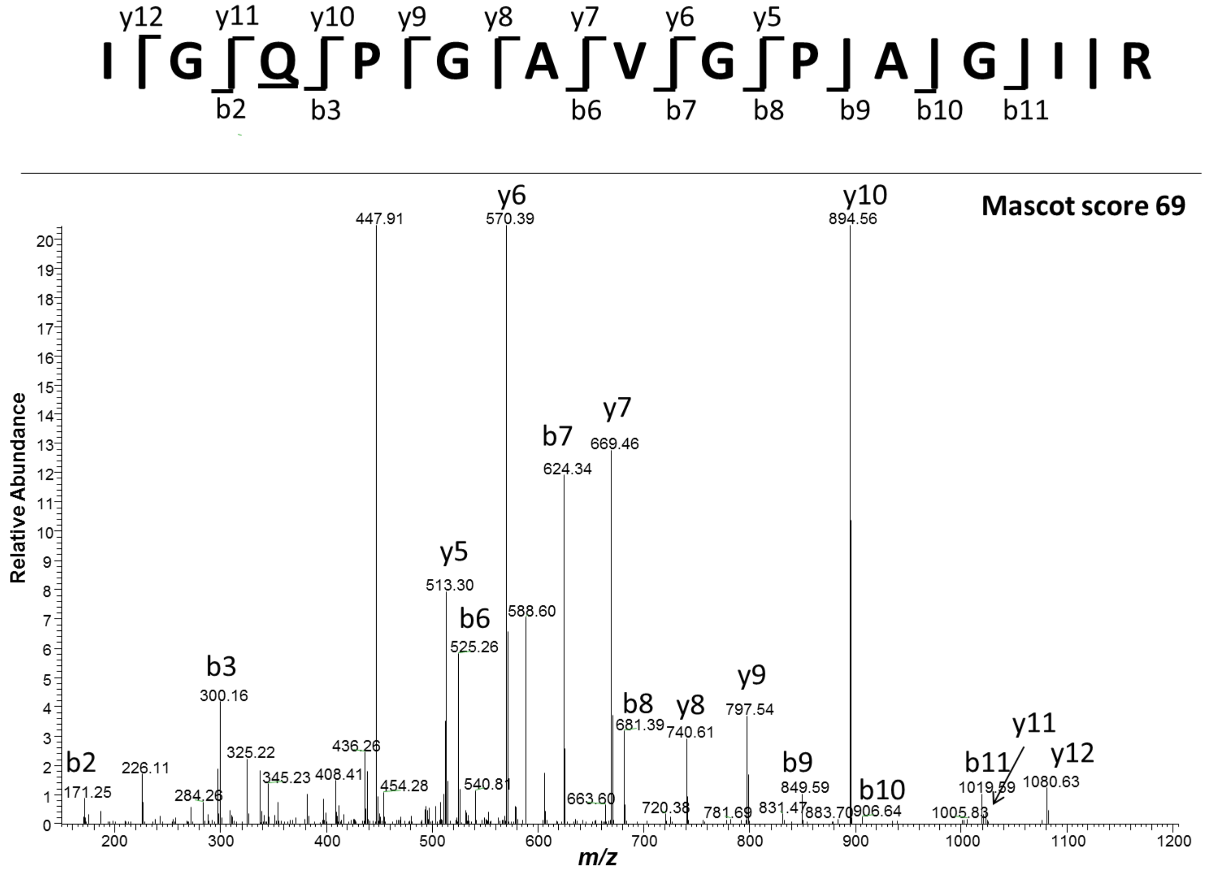

Figure S5: Tandem MS spectrum of IGQPGAVGPAGIR, M=1192.656, with deamidation in Q_3 from collagen alpha-2(I) chain CO1A2_BOVIN, identified by Mascot, in the flake sample NH2824.

NH2825: tandem MS spectra, charybdin

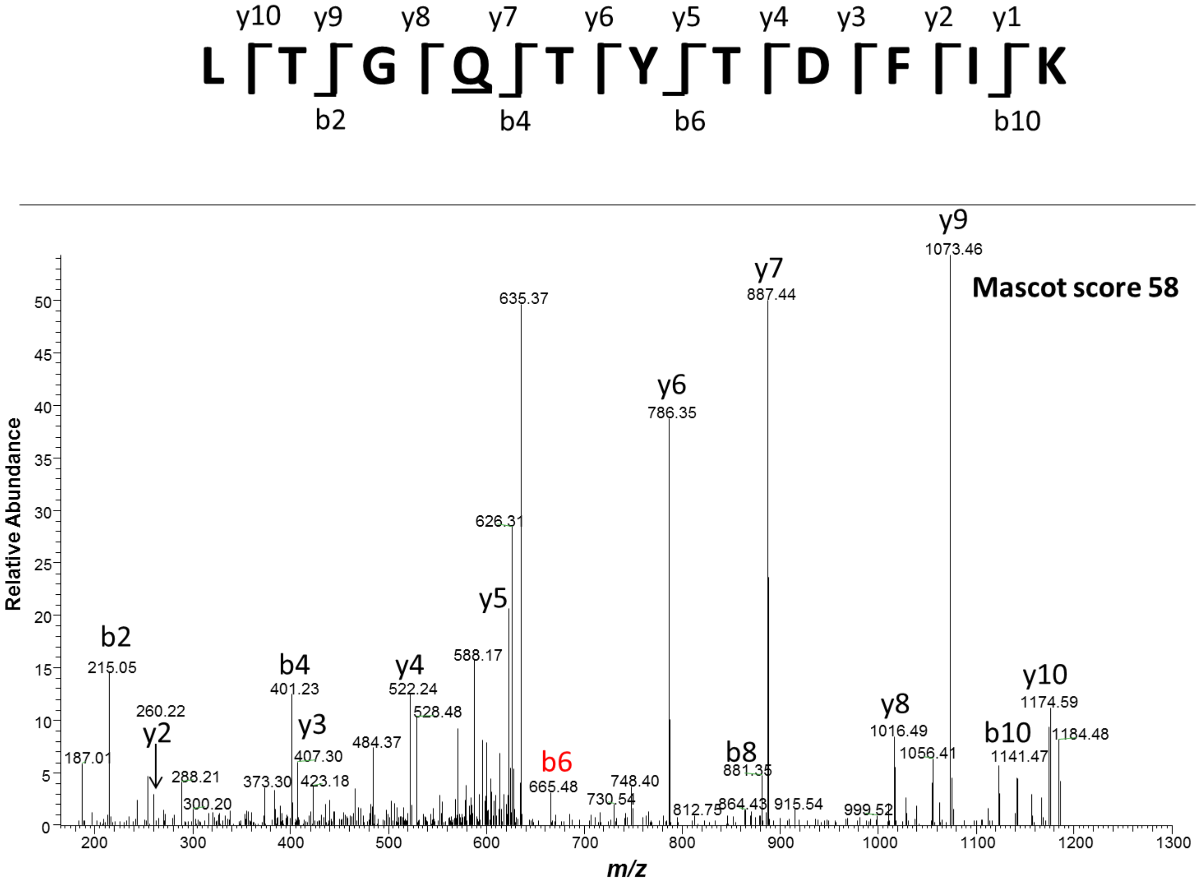

Figure S6: Tandem MS spectrum of LTGQTYTDFIK, M=1286.639, with deamidation in Q_4 from ribosome-inactivating protein charybdin RIP_DRIMA, identified by Mascot (in red are fragments manually identified), in basket sample NH2825.

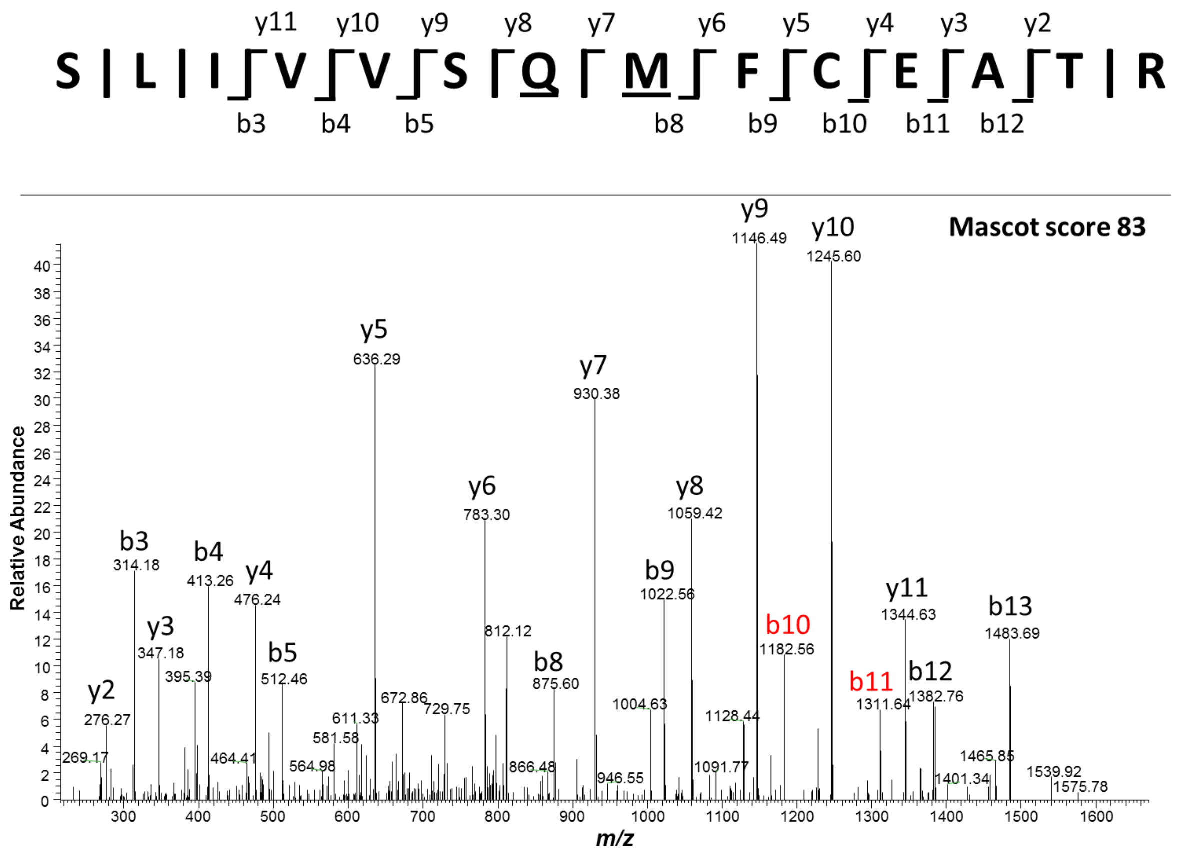

Figure S7: Tandem MS spectrum of SLIVVSQMFCEATR, M=1656.788, with deamidation in Q_7 and oxidation in M_8 from ribosome-inactivating protein charybdin RIP_DRIMA, identified by Mascot (in red are fragments manually identified), in basket sample NH2825.

Table S5: Proteomic results on the skull samples **NH2968** and **NH2969**: Protein name, accession number, species, #peptides (#peptides > mascot score 40), percentage coverages, ion score (ion score > mascot score 40) and role of the protein

Protein	Acc. No.	Species	# pep. (>40)	Cov. (%)	Ion Score (>40)	Function and location
NH2968<br>Skull (background)						
Keratin, type II cytoskeletal 1	K2C1_HUMAN	*Homo sapiens*	36(9)	39	450(351)	Structural, skin
Keratin, type I cytoskeletal 16	K1C16_HUMAN	*Homo sapiens*	32(9)	49	358(336)	Structural, skin
Keratin, type I cytoskeletal 9	K1C9_HUMAN	*Homo sapiens*	24(5)	32	223(157)	Structural, skin
Lactotransferrin	TRFL_HUMAN	*Homo sapiens*	18(3)	29	166(120)	Glycoprotein
Serum albumin	ALBU_HUMAN	*Homo sapiens*	24(3)	31	106(85)	Maintain oncotic pressure and transport protein<br>Plasma
NH2969<br>Skull (net pattern)						
Keratin, type II cytoskeletal 1	K2C1_HUMAN multiple	*Homo sapiens*	21(9)	34	241(233)	Structural, skin

Most proteins identified are from Human and most likely contamination. The cytoskeletal keratins identified are common laboratory contamination from dead skin resulting from handling. An indication that the proteins are likely false positives is the complete absence of Asn and Gln deamidation (a modification commonly identified in ancient proteins) in the proteins identified in the skull while deamidation is consistently found in the proteins identified in the basket.

% of lipid extract

Table S6: Sample amounts and % of lipid extract obtained after solvent extraction (CH_2Cl_2/CH_3OH, 1/1, v/v).

Sub-sample number	Sample amounts for lipid analysis (mg)	Lipid extract (%)
**NH2824**	483	< 1
**NH2825**	22	3
**NH2826**	54	9
**NH2968**	22	67
**NH2969**	52	>70

Total lipid extracts analysed by GC/MS

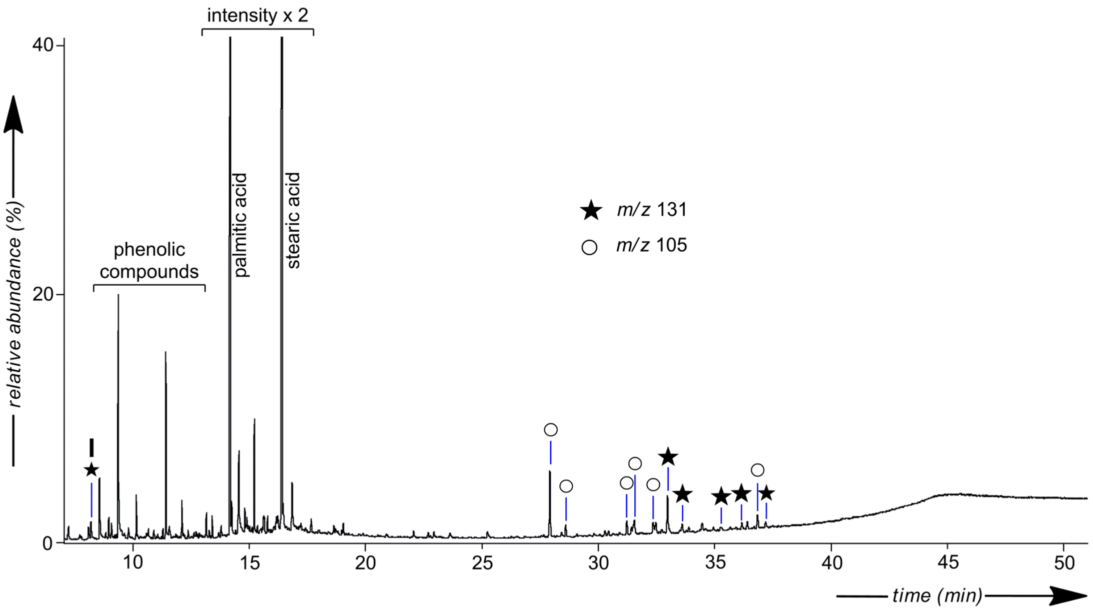

Figure S8: Gas chromatogram of the total lipid extract from the Neolithic skull (NH2969) found in the Nahal Hemar cave. (I) cinnamic acid. Cinnamic (*m/z* 131) and benzoic (*m/z* 105) ester derivatives are identified by filled stars and open circles, respectively. Alcohols are detected as acetate derivatives and acids as methyl esters.

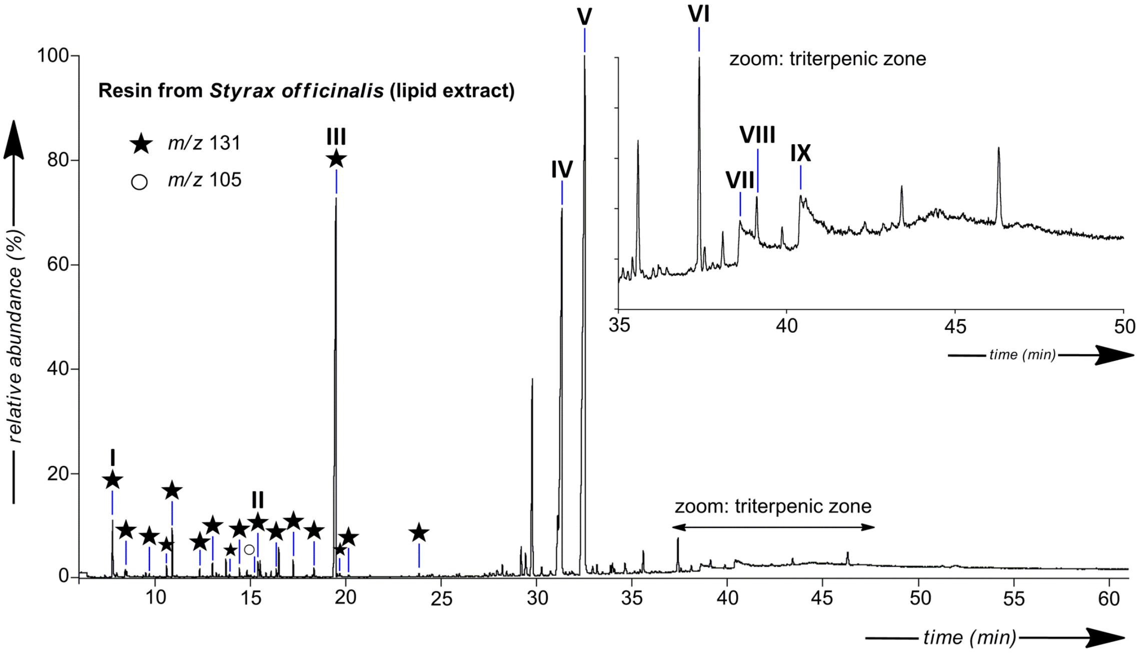

Figure S9: Gas chromatogram of the total lipid extract of a fresh resin sample of *Styrax officinalis* (*Styracaceae*). Cinnamic (*m/z* 131) and benzoic (*m/z* 105) ester derivatives are identified respectively by filled stars and open circles. Alcohols are detected as acetate derivatives and acids as methyl esters. Numbers refer to the compound structures reported on Fig. 5.

Identification of the triterpenoids occurring in samples NH2968, NH2969 and in the resin of *S. officinalis*

The structural identification of the triterpenoids as C-6 oxygenated derivatives of oleanolic acid in samples NH2968 and NH2969 and in the fresh and oxidized resin of *S. officinalis* is mainly based on mass spectral investigations using GC-MS (electron impact (EI), chemical ionization (CI) and field ionization (FI) modes) as well as on comparison with published mass spectra of triterpenoids from *Styrax sp*[10] or other plant species[11] and of amyrin-related epoxylactones[12].

*Identification of compounds **VII** to **IX***

The EI mass spectra of compounds **VII** - **IX** (Fig. S10) display a characteristic fragment at *m/z* 262 corresponding to the Retro-Diels Alder fragmentation of olean-12-ene derivatives bearing a carbomethoxy group at C-17[13] and a fragment at *m/z* 203 resulting from the loss of the carbomethoxy function. GC-MS analysis with FI mode allowed the molecular weight of compounds **VII**, **VIII** and **IX** to be unambiguously determined ($M^{+\cdot}$ 484, 526 and 528, respectively). The occurrence of a non-acetylated hydroxyl group on the *A*/*B* ring moiety of compounds **VII** and **IX** is evidenced by the presence of a fragment at *m/z* 466 and 450, respectively, resulting from the loss of 18 Da ($-H_2O$) in the mass spectra (Fig. S10). In this respect, the high steric hindrance around the 6β-hydroxyl group prevented it from being acetylated. Lastly, comparison of the mass spectra of **VII**, **VIII** and **IX** (Fig. S10) with spectra published in the literature[2] firmly supports identification of these compounds as methyl 3-oxo-6β-hydroxy-olean-12-en-28-oate, methyl 3β-acetoxy-6-oxo-olean-12-en-28-oate, and methyl 3β-acetoxy-6-hydroxy-olean-12-en-28-oate, respectively.

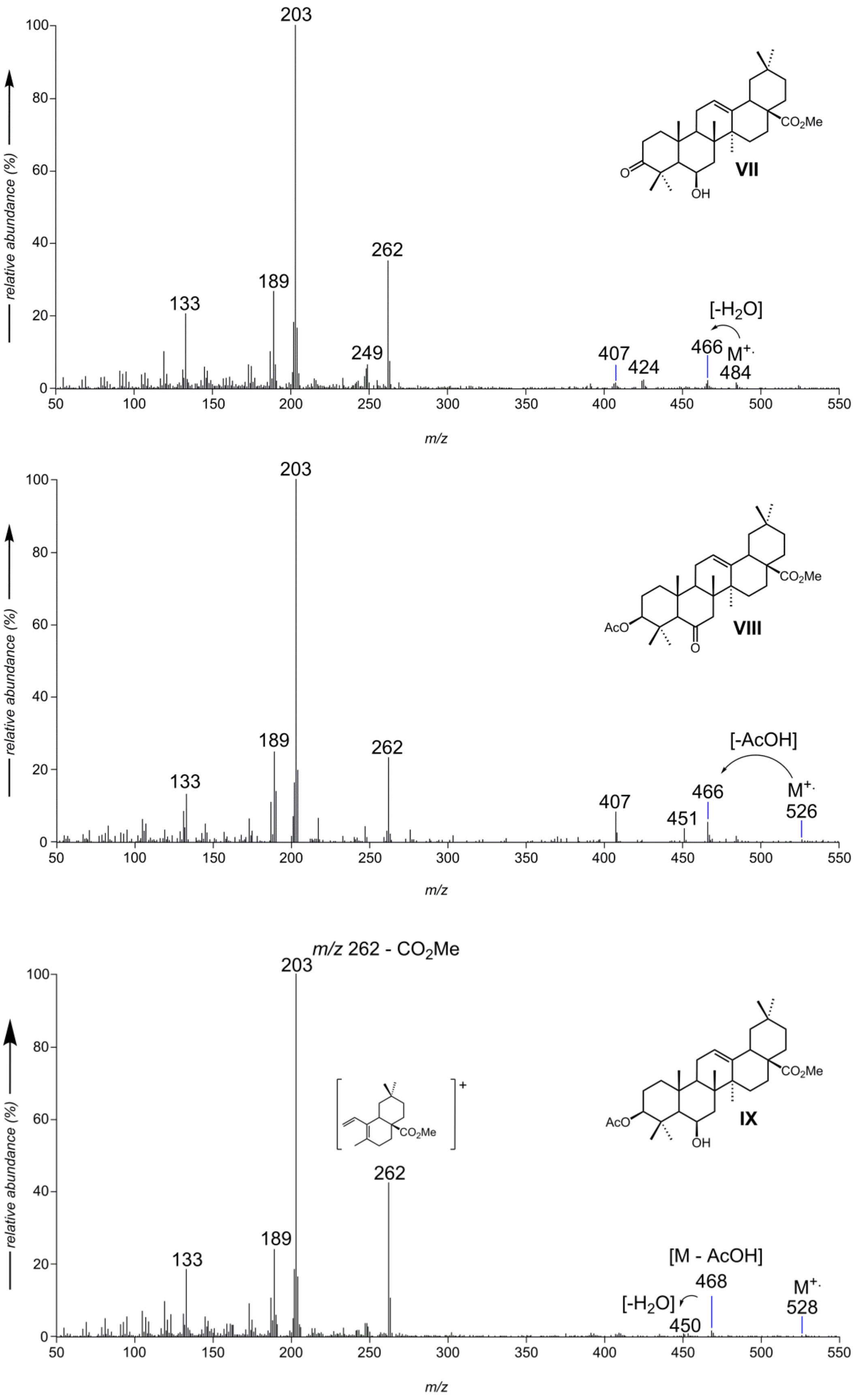

Fig S10: Mass spectra of compounds **VII - IX** (EI, 70 eV)

*Identification of compounds **XII** to **XIV***

Compounds **XII**, **XIII** and **XIV** were identified as the epoxylactone analogues of the triterpenoids **VII, VIII** and **IX**. Indeed, compounds **XII** to **XIV**, found both in the archaeological sample NH2968 and in the resin of *S. officinalis* after the laboratory oxidation experiment, display mass spectra with a fragmentation pattern very similar to that described in the literature for amyrin-derived epoxylactones[10,12,14]. The mass spectra of **XII** - **XIV** share common main fragments at *m/z* 189, 204 and 217 as well as fragments at *m/z* 291, 249 and 293, respectively, for **XII**, **XIII** and **XIV** which are interpreted as characteristic rearrangements fragments of amyrin-related 11α-12α-epoxylactones by analogy with the fragmentation pattern proposed by Kamiya et *al.*[14]. Furthermore, unambiguous determination of the molecular weight by mass spectrometry in FI mode ($M^{+\cdot}$526, 484 and 528, respectively) and the similarity of the EI mass spectra of compounds **XII** - **XIV** with those published in the literature[10] further support the identification of compounds **XII** - **XIV** as the epoxylactone analogues of the C-6 oxygenated oleanolic acid derivatives occurring in fresh resin of *S. officinalis*.

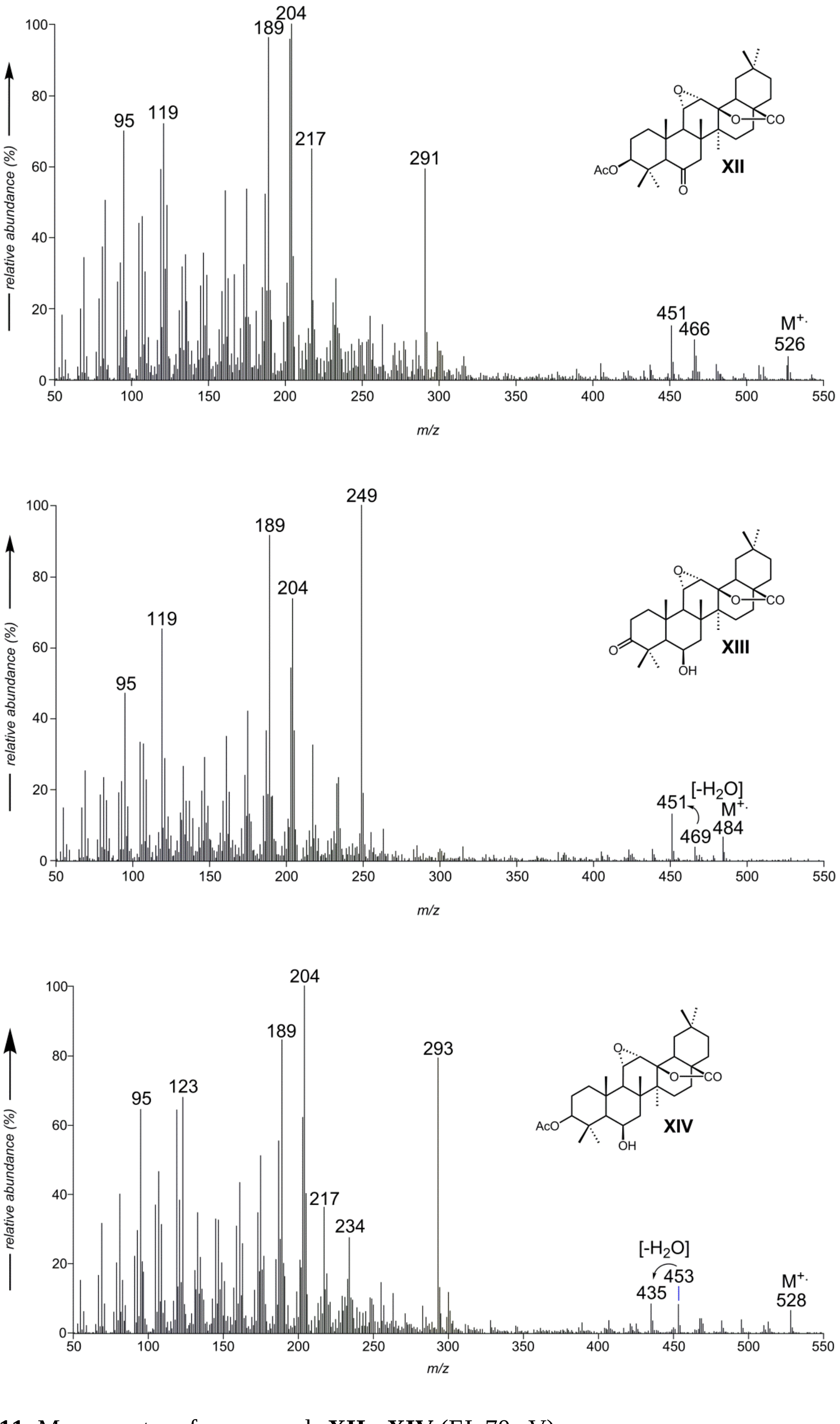

Fig S11: Mass spectra of compounds **XII - XIV** (EI, 70 eV).

Taxonomy of *Charybdis maritima* (*Drimia maritima*)

The World Checklist of Selected Plant Families (http://apps.kew.org/wcsp/qsearch.do) indicates the first mention of the sea squill under the name "*Scilla maritima"* in 1753, then later as "*Urginea maritima* (L.) Baker, J. Linn. Soc., Bot. 13: 221 (1873)", "*Drimia maritima* (L.) Stearn, Ann. Mus. Goulandris 4: 204 (1978)", and finally "*Charybdis maritima* (L.) Speta, Phyton (Horn) 38: 60 (1998)". There are 12 species under the genus Charybdis which are commonly accepted under their synonyms in the Drimia genus, under which the World Checklist of Selected Plant Families gives 181 records (at time of publication). The number of species recorded in NCBI under the genus Drimia is of 66.

Drimia belongs to the Urgineoideae subfamily of the Hyacinthaceae family (hyacinth family) which contains three other subfamilies (Hyacinthoideae, Ornithogaloideae and Oziroeoideae). According to Pfosser and Speta (2004)[15], the sea onion, well known since Antiquity for its medicinal properties and by the Romans as Scilla, was first wrongly attributed to the Scilla genus in the Hyacinthoideae subfamily. The genus Urginea (Urgineoideae subfamily) was created in the 1830s and was followed by the inclusion by Baker of the sea onion in 1873. The genera *Bowiea* and *Drimia* were then added and *Charybdis* created later, in which these authors (Pfosser and Speta (2004)[15]) have classified the Mediterranean squills.

Charybdin

Charybdin was characterised and named by Touloupakis et al. (2006)[16] as a novel 29 kDa type I ribosome-inactivating protein (RIP) found in bulbs of *C. maritima*. There are seven other ribosome-inactivating proteins characterized in the Hyacinthaceae family; of them the closest species has less than 50% percentage coverage in common with charybdin.

RIPs inhibit protein synthesis by enzymatically damaging ribosomes; there are of two types, type I has a single peptide chain, type II contains two. By cleaving a certain site in rRNA, they stop protein synthesis, thus inhibiting ribosome activity[17]. Some RIPs such as ricin are potent toxins. In *C. maritima*, Touloupakis et al.[16] mention that with 150-200 mg per 100 g, the "bulbs contain extremely high quantities of the charybdin protein. The initial extract contained mainly charybdin and very small amounts of other proteins, which were only observed when the gel was overloaded." Charybdin was the only protein characterised in this study. At the time of writing, there are 265 entries available in NCBI (http://www.ncbi.nlm.nih.gov/) for the Urgineoideae sub-family, 174 entries for the genus Drimia/Charybdis and 11 for *D. maritima* (mostly chloroplast proteins).

In addition to characterising charybdin, the study highlights a substitution at position 79 in the active site of RIPs where tyrosine is replaced in charybdin by valine. The authors argue that this substitution might be responsible for its low inhibitory activity compared with other RIPs, and that the protein (as the main protein constituent in the bulb) might have a different function such as storage. The peptide which bears Val79, DDL**V**LR, was successfully identified with a score of 49, as well as IHRDDL**V**LR with a score of 42. While the first peptide is highly conserved in plants and bacteria (but not identified in RIPs), the second one is found only in a handful of bacterial species.

Amino acid distribution in charybdin and collagen

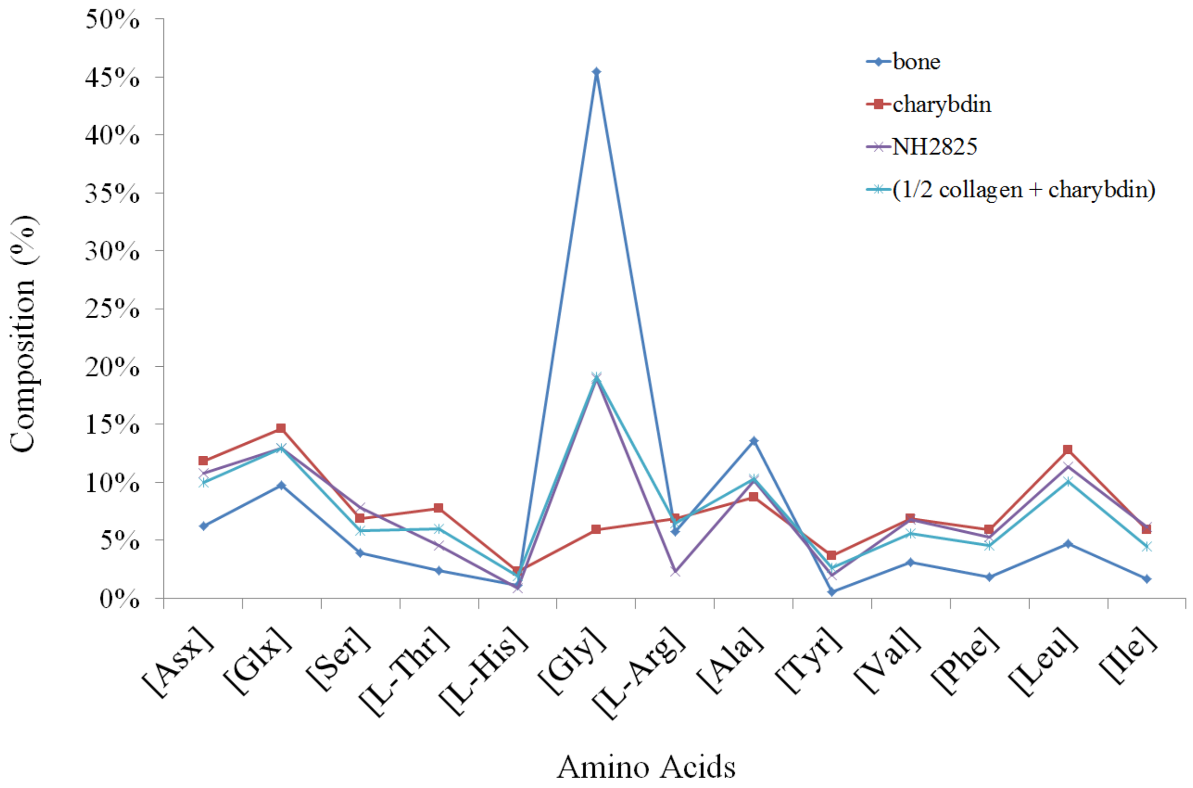

Figure S12: The theoretical distribution in amino acids (relative distribution of the amino acids identified by RP-HPLC) in the charybdin protein from *Charybdys maritima* (RIP_DRIMA) is plotted against the amino acid analysis distribution obtained in the bone reference and sample NH2825. In the hypothesis that charybdin is the sole plant protein constituent in the organic residue of NH2825, it would require twice as much charybdin as collagen to bring the concentration in glycine from its level in bone collagen to the one detected in NH2825.

References

1 Bar-Yosef, O. *A cave in the desert: Nahal Hemar 9000-year old-finds*. Vol. 258 (The Israel Museum, 1985).

2 Bar-Yosef, O. & Alon, D. Nahal Hemar cave. The excavations. *'Atiqot* **18**, 1-30 (1988).

3 Stankiewicz, B. A., Hutchins, J. C., Thomson, R., Briggs, D. E. G. & Evershed, R. P. Assessment of bog-body tissue preservation by pyrolysis-gas chromatography/mass spectrometry. *Rapid Commun Mass Spectrom* **11**, 1884-1890 (1997).

4 Chiavari, G. & Galletti, G. C. Pyrolysis—gas chromatography/mass spectrometry of amino acids. *J. Anal. Appl. Pyrolysis* **24**, 123-137 (1992).

5 Stankiewicz, A. B. *et al.* Recognition of Chitin and Proteins in Invertebrate Cuticles Using Analytical Pyrolysis/Gas Chromatography and Pyrolysis/Gas Chromatography/Mass Spectrometry. *Rapid Commun Mass Spectrom* **10**, 1747-1757 (1996).

6 Branca, C., Giudicianni, P. & Di Blasi, C. GC/MS Characterization of Liquids Generated from Low-Temperature Pyrolysis of Wood. *Ind. Eng. Chem. Res.* **42**, 3190-3202 (2003).

7 Custódio, D. L. & Veiga-Junior, V. F. True and common balsams. *Revista Brasileira de Farmacognosia* **22**, 1372-1383 (2012).

8 Modugno, F., Ribechini, E. & Colombini, M. P. Aromatic resin characterisation by gas chromatography–mass spectrometry: Raw and archaeological materials. *J Chromatogr A* **1134**, 298-304 (2006).

9 Hovaneissian, M., Archier, P., Mathe, C. & Vieillescazes, C. Contribution de la chimie analytique à l'étude des exsudats végétaux styrax, storax et benjoin. *C R Chim* **9**, 1192-1202 (2006).

10 Wang, F., Hua, H., Pei, Y., Chen, D. & Jing, Y. Triterpenoids from the resin of *Styrax tonkinensis* and their antiproliferative and differentiation effects in human leukemia HL-60 cells. *J. Nat. Prod.* **69**, 807-810 (2006).

11 Wahlberg, I. & Enzell, C. 3-oxo-6β-hydroxyolean-12-en-28-oic acid, a new triterpenoid from commercial tolu balsam. *Acta Chemica Scandinavica* **25**, 70-76 (1971).

12 Majumder, P. L. & Bagchi, A. Oxidative transformations of triterpenoids of the ursane and oleanane skeleta with hydrogen peroxide. introduction of 11,12-double bond and 13(28)oxide moiety in the ursane system. *Tetrahedron* **39**, 649-655 (1983).

13 Budzikiewicz, H., Wilson, J. M. & Djerassi, C. Mass Spectrometry in Structural and Stereochemical Problems. XXXII.1 Pentacyclic Triterpenes. *Journal of the American Chemical Society* **85**, 3688-3699 (1963).

14 Kamiya, K., Yoshioka, K., Saiki, Y., Ikuta, A. & Satake, T. Triterpenoids and flavonoids from *Paeonia lactiflora*. *Phytochemistry* **44**, 141-144 (1997).

15 Pfosser, M. F. & Speta, F. From *Scylla* to *Charybdis* - is our voyage safer now? *Plant Syst. Evol.* **246**, 245-263 (2004).

16 Touloupakis, E. *et al.* Isolation, characterization, sequencing and crystal structure of charybdin, a type 1 ribosome-inactivating protein from Charybdis maritima agg. *FEBS J.* **273**, 2684-2692 (2006).

17 Stirpe, F. in *Molecular Neurosurgery With Targeted Toxins* (eds Ronald G. Wiley & Douglas A. Lappi) 9-29 (Humana Press, 2005).
